# Environment-Dependent Dimerization as a Functional Switch in Leech Cystatin CysHv

**DOI:** 10.3390/toxins18070300

**Published:** 2026-07-10

**Authors:** Melissa Regina Fessel, Ana Marisa Chudzinski-Tavassi, Fernanda Faria

**Affiliations:** 1 Laboratory of Development and Innovation, Butantan Institute, Av. Vital Brasil, 1500-Butantã, Sao Paulo 05503-900, Brazil; melissa.fessel@butantan.gov.br (M.R.F.); ana.chudzinski@butantan.gov.br (A.M.C.-T.); 2 Centre of Excellence in New Target Discovery-CENTD, Butantan Institute, Av. Vital Brasil, 1500-Butantã, Sao Paulo 05503-900, Brazil

**Keywords:** CysHv, cystatin, domain swap dimerization, leech, control, inhibitory activity

## Abstract

Cystatins from hematophagous organisms modulate host proteases and are promising biotechnological tools. CysHv, a secreted cystatin from the Brazilian leech *Haementeria vizottoi*, was previously characterized as a potent inhibitor of papain and cathepsin L. In this work, it is shown that its inhibitory activity is tightly linked to conformational state. CysHv is purified as an active monomer but forms inactive dimers in a time-, temperature-, and concentration-dependent manner. A domain-swapping-like mechanism was hypothesized, and a disulfide-engineered mutant restricting local flexibility remained predominantly monomeric, supporting the role of conformational plasticity. Dimer formation correlates with loss of inhibitory activity and is accelerated at acidic pH, suggesting that pH modulates the kinetics rather than triggering dimerization. Comparative structural analysis of cystatins from hematophagous organisms revealed recurrent pairs of oppositely charged residues in β-strands flanking the inhibitory loop in secreted cystatins, but these pairs are generally absent in intracellular homologs. Although causality cannot be established and additional determinants likely contribute to dimerization, this pattern is consistent with pH-sensitive modulation of conformational behavior. Together, these findings identify CysHv as a dimer-prone cystatin for which oligomerization, potentially via a domain-swapping-like mechanism, could act as an environment-dependent regulator of inhibitory activity.

## 1. Introduction

Biodiversity harbors a vast reservoir of molecules with potential biotechnological and therapeutic applications, well represented by the plant-derived diterpenoid paclitaxel, used as a chemotherapy drug [1,2]; captopril, employed in human hypertension treatment and developed based on the template of a bradykinin-potentiating peptide first identified in the venom of *Bothrops jararaca* [3,4]; and hirudin, the principal anticoagulant (a thrombin inhibitor) of the medicinal leech *Hirudo medicinalis*, clinically used as the treatment of choice for heparin-induced thrombocytopenia [5,6]. Within this diversity, organisms with specialized lifestyles, such as hematophagy, have evolved unique proteins as adaptive strategies to modulate host physiology, counteract defenses, and ensure successful feeding and survival [7,8].

Among the various proteins produced by hematophagous organisms, protease inhibitors are particularly important, as they target host proteolytic cascades and regulate processes such as hemostasis, inflammation, and immunity, enabling successful blood feeding and digestion. Salivary secretions of blood-feeding animals are especially enriched in these inhibitors, making them attractive for biotechnological and therapeutic exploration [8,9,10].

In this context, transcriptomic analyses of the Brazilian leech *Haementeria vizottoi* salivary complexes [11] identified a cystatin-like transcript that was recombinantly expressed and biochemically characterized. This protein, herein named CysHv, is a potent inhibitor of cysteine proteases such as cathepsin L and papain [12], suggesting that it may modulate proteolytic processes associated with the parasitic lifestyle.

The cystatin superfamily, widely distributed across all domains of life [13], comprises tight-binding reversible cysteine protease inhibitors that play key roles in regulating protein degradation [14,15]. Cystatins are classified under family I25 in the MEROPS database (https://www.ebi.ac.uk/merops/cgi-bin/famsum?family=I25 (accessed on 20 May 2026)) and primarily inhibit cysteine proteases of the papain family (C1) and, in some cases, the legumain family (C13) [16,17]. They share a conserved structural fold, consisting of a β-sheet wrapped around a central α-helix, and a wedge-shaped tripartite inhibitory interface formed by an N-terminal glycine and two ß-hairpin loops (L1 and L2) at the extremity of consecutive β-strands, that engages papain-like (C1) proteases [14,18,19]. Targeting legumain (C13) proteases is mediated by an additional loop at the opposite extremity of the β-sheet [16,17,18].

Cystatins were initially classified based on sequence similarity, disulfide bond content, and cellular localization into three families: single domain intracellular stefins (type 1), single domain extracellular cystatins (type 2), and multi-domain extracellular kininogens (type 3) [20]. The MEROPS database now provides a more comprehensive, structure-informed classification that groups cystatins into the I25 family, further subdivided into I25A, I25B, I25C, and unclassified subfamilies [21]. Focusing on single-domain cystatins, intracellular stefins (I25A) are small (~11 kDa), generally lack a signal peptide, disulfide bonds, and carbohydrate content [14,20,22]. Extracellular cystatins (I25B) are slightly larger (~13–14 kDa), usually secreted, generally lack glycosylation, and contain two conserved disulfide bonds [14,22].

Based on the canonical numbering of human cystatin C, these covalent linkages are formed by four specific cysteine residues, establishing pairs between Cys73-Cys83 and Cys97-Cys117 [23]. This covalent organization is highly conserved in the corresponding positions of family members, forming an essential signature of the cystatin fold [22,24]. Functionally, these disulfide bonds do not participate directly in target protease inhibition, as the inhibitory mechanism relies on the rigid wedges formed by the N-terminus and loops L1 and L2 [14,22]. Instead, they play a crucial role in restricting conformational flexibility, thereby providing global structural integrity and thermodynamic stability to the protein [14]. Ultimately, these distinctive biochemical features distinguish intracellular inhibitors from their secreted counterparts. These structural and localization differences reflect distinct biological roles [9,14,25,26].

Cystatins from hematophagous organisms have been extensively studied. In these organisms, cystatins are present in saliva and a variety of other tissues, contributing to parasite survival and successful blood feeding [27,28,29,30,31,32,33,34,35,36,37,38,39,40,41,42,43,44,45]. Despite sharing a conserved fold, these proteins can display distinct functional behaviors depending on their environmental context [9,14,25,26]. Several hematophagous cystatins with distinct inhibitory profiles have been reported over the years, encompassing a range of activities, including anti-inflammatory, immunosuppressive, immunomodulatory, and bacteriostatic effects [28,29,33,34,38,39,40,41,45].

An additional level of structural complexity in cystatins arises from domain swapping, a phenomenon in which identical protein monomers exchange structural elements to form oligomers [46,47,48]. In some cystatins, including human cystatin C (hCC), a classic example of the phenomenon [23], domain-swapping dimerization has been observed to involve extension of the L1 loop, which adopts a β-strand-like conformation, allowing exchange of the α-helix and adjacent β-strands between protomers. This process disrupts the canonical inhibitory interface, is often associated with kinetically stable oligomer formation, and can be influenced by environmental factors such as pH and temperature [49,50,51]. In hCC, domain swapping has been linked to hereditary amyloid disease [23,52,53], and it can be suppressed by an engineered disulfide bond that stabilizes the monomeric fold [54].

While domain swapping is well-characterized in vertebrates [17,23,55], structural studies of invertebrate cystatins indicate that domain swapping can occur, although only a few cases have been documented to date [33,56,57,58,59], structural studies of invertebrate cystatins indicate that domain swapping can occur, although only a few cases have been documented to date. Among the numerous invertebrate cystatins described, the documented domain-swapped dimers include the tick salivary protein Sialostatin L, which crystallizes as a dimer [33]; another secreted tick cystatin, Amacstatin 2, which also forms a domain-swapped dimer, although its functional characterization is not yet reported (PDB 8r29); and EmCystatin-B, a stefin from the tissue parasite *Echinococcus multilocularis* that undergoes domain swapping followed by tetramerization stabilized by a disulfide bond [58]. These observations highlight that domain swapping in invertebrate cystatins is a mechanistically accessible phenomenon but still largely unexplored.

Despite the breadth of structural knowledge in vertebrate cystatins and the initial invertebrate crystal structures, comprehensive analyses of conformational dynamics and oligomerization in hematophagous cystatins under physiologically relevant conditions in solution remain limited. For example, Sialostatin L crystallizes as a dimer but is predominantly monomeric in solution [33], indicating that protein oligomerization and conformational equilibria can be strongly influenced by environmental factors, which are generally not systematically explored in initial protein characterization.

Addressing this knowledge gap, the present work investigates the conformational and functional behavior of CysHv in solution, focusing on the relationship between oligomerization, environmental conditions, and inhibitory activity. CysHv is characterized here as a dimer-prone cystatin, and the influence of factors such as pH and temperature on domain-swapped-like dimerization is examined, providing the first detailed demonstration of this phenomenon in solution for a hematophagous cystatin. Understanding how environmental conditions modulate cystatin structure and function not only advances the structural biology of the cystatin superfamily but also has important implications for functional characterization, both for elucidating their biological roles and for supporting future development of biotechnological applications of protease inhibitors identified through biodiversity-driven explorations.

## 2. Results

### 2.1. CysHv Is Obtained as a Well-Structured Active Monomer

**Figure 1:**
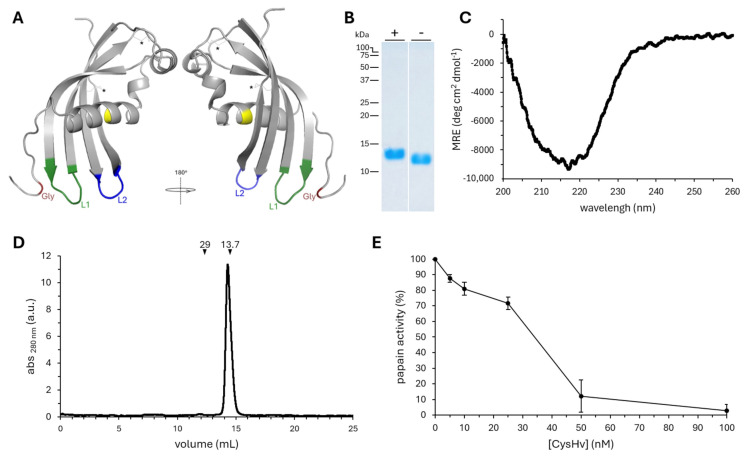
Structural prediction, recombinant expression, characterization, and functional analysis of CysHv. (**A**) Two poses of the CysHv Robetta-predicted 3D structure model are represented in a cartoon view, indicating the predicted disulfide bridges (lines, *), the predicted unpaired Cys residue (yellow), and the inhibitory triad: N-terminal Gly residue (red), loop L1 (green), and loop L2 (blue). (**B**) Purity assessment by SDS-PAGE 15%, under reducing (+) and non-reducing conditions (−), stained with Coomassie blue. On the left, molecular mass standards, in kDa. (**C**) Circular dichroism spectrum at far UV, showing molar residual ellipticity (MRE) as a function of wavelength. (**D**) Evaluation of freshly purified CysHv oligomeric state by SEC. The graph shows absorbance (abs) at 280 nm, in arbitrary units (a.u.), as a function of the elution volume. On top, the retention volumes of commercial standard proteins are indicated in kDa. (**E**) CysHv concentration curve on papain inhibition. Papain (10 nM) activity was evaluated at 30 °C in the presence of the substrate Z-FR-MCA (200 μM) and the indicated concentrations of CysHv. In (**E**), data represent the mean of three independent experiments ± standard deviation (SD).

CysHv was previously identified in the transcriptome of salivary complexes of the leech *H. vizottoi* [11] as an extracellular cystatin, and it was produced recombinantly by our group as a secreted protein in *Pichia pastoris*, where it was purified and characterized as a potent inhibitor of cathepsin L and papain [12]. The mature protein has an approximate molecular mass of 12.3 kDa, and its corresponding theoretical three-dimensional model was generated using the Robetta server to provide an initial visualization of its structural topology. Although these atomic coordinates remain predictive in the absence of high-resolution experimental structural data, the model displays the canonical cystatin fold, consisting of a β-sheet wrapped around a perpendicular α-helix. The conserved elements of the inhibitory triad are preserved, including the N-terminal glycine residue and the L1 (QVVAG) and L2 (PW) loops (Figure 1A). The analysis of the structural model also reveals a predicted unpaired, free cysteine residue located within the α-helix segment (Figure 1A).

To improve yield and purity, alternative purification strategies were evaluated. A combination of hydrophobic interaction, anion exchange, and size exclusion chromatography (Figure S1) resulted in a highly purified protein, as confirmed by Coomassie blue-stained SDS-PAGE under both reducing and non-reducing conditions, where CysHv migrated as a single band corresponding to its monomeric molecular mass (Figure 1B).

CysHv secondary structure was assessed by far-UV circular dichroism (CD) spectroscopy, revealing a well-folded protein (Figure 1C). To estimate the secondary structure composition of CysHv, the experimental CD spectrum was deconvoluted using BeStSel, yielding 39.8% β-strand, 11.9% α-helix, 10.0% turn, and 38.3% other structures. According to DSSP analysis of the predicted model, this structure comprises 49.1% β-strand, 16.1% α-helix, 8.0% turn, and 26.8% other structures, which include coils/loops (15.2%), bends (5.4%), and 3_10_-helix (6.2%). The experimental CD data qualitatively agree with the computational model, indicating that the structural content is consistent with a predominantly α + β protein fold characteristic of the cystatin superfamily.

Freshly purified CysHv (0.4 g/L) eluted as a monomer by size exclusion chromatography (SEC) in purification buffer (20 mM Tris-HCl, pH 8.0, 50 mM NaCl, 1 mM EDTA) on a Superdex 75 10/300 column (Figure 1D).

CysHv displayed dose-dependent inhibitory activity against the model cysteine protease papain, with 50 nM CysHv inhibiting approximately 90% of the activity of the 10 nM enzyme (Figure 1E).

### 2.2. CysHv Can Adopt Distinct Oligomeric and Functional States

**Figure 2:**
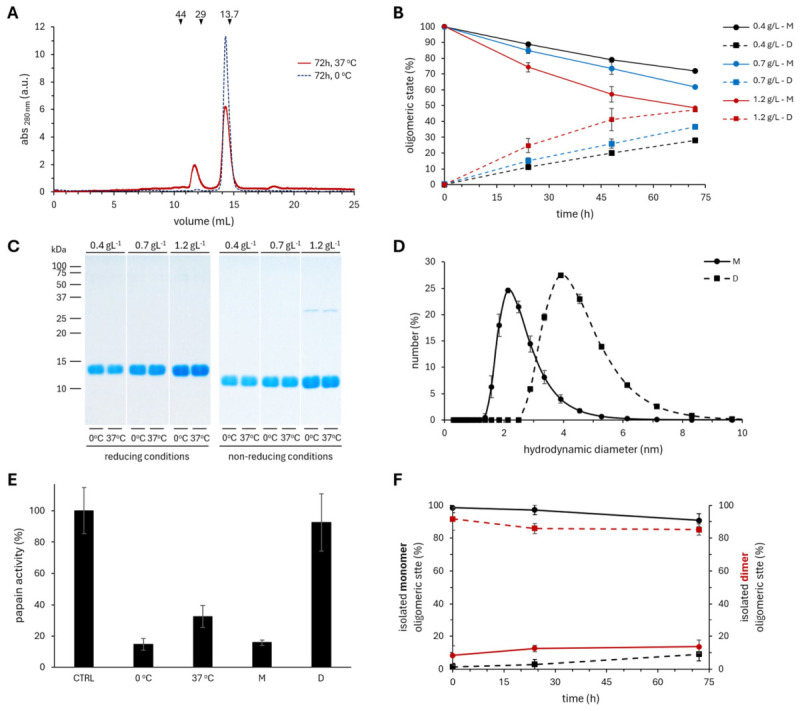
Monomer CysHv undergoes time-, temperature-, and concentration-dependent dimerization, with functional consequences. (**A**) Freshly purified CysHv (0.4 g/L, pH 8) was incubated at the indicated conditions and analyzed by SEC. On top are retention volumes of standard proteins in kDa. (**B**) Samples with increasing concentrations of CysHv were incubated at 37 °C, and the resulting oligomeric state distribution was analyzed over time by SEC. Monomer (M) and dimer (D) content (%) were determined from the chromatograms. (**C**) SDS-PAGE (15%) analysis of CysHv incubated for 72 h at different concentrations (0.4, 0.7, and 1.2 g/L) and temperatures (0 °C and 37 °C). Samples were analyzed under reducing or non-reducing conditions, as indicated. (**D**) Isolated monomer and dimer of CysHv, obtained by SEC after thermal stress (1.2 g/L, 48 h, 37 °C, pH 8), were analyzed by DLS. (**E**) Papain (10 nM) activity was tested in the absence (CTRL) or in the presence of 50 nM CysHv treated for 48 h at the indicated temperatures (0 °C or 37 °C) or 50 nM isolated monomer or dimer (M and D, respectively). (**F**) Isolated monomeric (black) and dimeric (red) species were further incubated at 37 °C (pH 8), analyzed by SEC, and the resultant monomer (solid circles, solid lines) and dimer (solid squares, dashed lines) content were determined over time. Except for (**A**,**F**), which show representative results. Data are presented as the mean ± standard deviation (SD) of three independent experiments.

As part of the optimization of the CysHv purification, protein stability was evaluated by SEC under different conditions. CysHv (0.4 g/L) incubated in ice bath, at 0 °C, remained monomeric over 72 h, whereas incubation at 37 °C led to the appearance of a higher-molecular-mass species, consistent with dimer formation (Figure 2A).

Time-course analysis at 37 °C across different protein concentrations showed progressive dimer accumulation, enhanced at higher protein concentrations, indicating a time- and concentration-dependent process (Figure 2B).

Considering the presence of an unpaired cysteine residue in CysHv revealed by the analysis of its computationally predicted structure (Figure 1A), the electrophoretic profile of CysHv subjected to thermal stress (0 °C and 37 °C for 72 h) at different concentrations (0.4, 0.7, and 1.2 g/L) was analyzed under both reducing conditions and non-reducing conditions to investigate the possibility that dimerization is driven by intermolecular disulfide bridges (Figure 2C). Under reducing conditions, CysHv migrated strictly as a single band of approximately 14 kDa across all tested concentrations and temperatures. Under non-reducing conditions, a similar monomeric migration profile was observed for samples at 0.4 and 0.7 g/L. At the highest protein concentration (1.2 g/L), the monomeric population remained the predominant species; however, a minor, discrete band corresponding to a dimeric subunit mass was detected (Figure 2C). Importantly, the intensity of this faint band remained identical at both 0 °C and 37 °C, demonstrating that formation of this minor fraction is not temperature-dependent and cannot, alone, explain the increase in the dimer content observed by SEC analysis under these specific parameters (Figure 2B). This static profile contrasts with the pronounced, concentration-dependent accumulation of the dimeric species observed during thermal stress at 37 °C by SEC analysis (Figure 2B). These observations demonstrate that the accumulation of the CysHv dimeric population occurs without a corresponding increase in disulfide-linked species.

Monomeric and dimeric species were isolated by SEC from samples (1.2 g/L) incubated at 37 °C for 72 h, and their hydrodynamic sizes were determined by dynamic light scattering (DLS), revealing populations centered at (3.00 ± 0.06) nm and (4.38 ± 0.04) nm, respectively (Figure 2D).

A partially dimerized sample containing ~20% dimer (obtained from CysHv 0.4 g/L incubated at 37 °C for 48 h) showed ~15% reduction in inhibitory activity, whereas the isolated monomer remained fully active, and the isolated dimer was inactive (Figure 2E).

Isolated monomeric and dimeric species, obtained by SEC fractionation, were incubated at 37 °C to assess their stability over time. Under these conditions, both species remained largely preserved over 72 h, with only minor changes in their relative distribution (Figure 2F). These observations indicate that, once formed and isolated, both conformational states are maintained over the experimental timescale, with only limited interconversion detected under the diluted conditions used in these assays.

### 2.3. A Domain-Swapping-Based Model Supports Rational Design of a Conformationally Constrained CysHv Mutant

**Figure 3:**
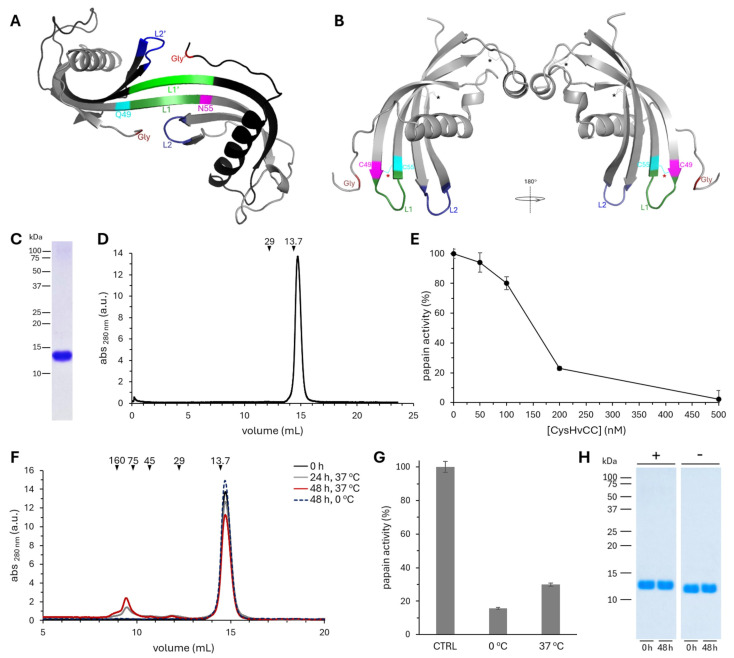
Rational design and characterization of a disulfide-engineered CysHv double mutant, CysHvCC. (**A**) CysHv structure prediction as a domain-swapped dimer (model Sialostatin L, PDB: 4zm8) is indicated (different chains in gray and black), highlighting elements of the inhibitory triad (Gly, disrupted L1 and L2 loops), and the residues marginal to L1, Q49, and N55. (**B**) CysHvCC predicted structure is shown in cartoon, indicating the inhibitory interface with intact L1 loop, and, in lines, the native (*) and the engineered (red *) disulfide bridges. (**C**) Purity assessment by SDS-PAGE 15%, Coomassie blue-stained. On the left, molecular mass standards in kDa can be seen. (**D**) Evaluation of freshly purified CysHvCC oligomeric state by SEC. The graph shows absorbance (abs) at 280 nm, in arbitrary units (a.u.), as a function of the elution volume. On top, the retention volumes of commercial standard proteins are indicated in kDa. (**E**) CysHvCC concentration curve on papain inhibition. Papain (10 nM) activity was evaluated at 30 °C in the presence of the substrate Z-FR-MCA (200 μM) and the indicated concentrations of CysHvCC. (**F**) Freshly purified CysHvCC (0.4 g/L, pH 8) was incubated at the indicated conditions and analyzed by SEC. On top are retention volumes of standard proteins in kDa. (**G**) Papain (10 nM) activity was tested in the absence (CTRL) or in the presence of 50 nM CysHvCC treated for 48 h at the indicated temperatures (0 °C or 37 °C). (**H**) SDS-PAGE (15%) analysis of CysHvCC incubated at 37 °C for 0 h and 48 h. Samples were analyzed under reducing (+) or non-reducing (−) conditions, as indicated. In (**E**,**G**), data are presented as the mean ± SD of three independent experiments.

To investigate the structural basis of CysHv dimerization, a domain-swapping model was generated based on the structure of the domain-swapped dimer of the homologous tick cystatin Sialostatin L (PDB: 4ZM8). In this model, the L1 loop extends and adopts a β-strand-like conformation, enabling the exchange of structural elements between protomers, including the α-helix and adjacent β-strands, while disrupting the canonical inhibitory interface (Figure 3A).

Stokes radii estimated from the structural models using US-SOMO yielded the predicted hydrodynamic diameter as 3.80 nm and 5.08 nm for monomeric and domain-swapped dimeric species, respectively. These values were consistent with DLS measurements for isolated monomeric and dimeric species (Figure 2D), supporting the plausibility of the domain-swapped model.

Considering this model, residues Q49 and N55 were selected for site-directed mutagenesis. A double cysteine mutant, named CysHvCC, was designed to introduce a disulfide bond near the L1-flanking region to restrict its conformational flexibility and prevent the structural rearrangement required for dimer formation (Figure 3B).

Based on the parental CysHv expression plasmid, pD912-AK/340, site-directed mutagenesis was used to generate the mutant construct, pD912-AK/CysHvCC, which was integrated into the *Pichia pastoris* X-33 genome to obtain the stable clone used in this study (Figure S2).

Following the same protocol used for the wild-type protein, CysHvCC was successfully expressed and purified to homogeneity (Figure S3) as confirmed by Coomassie blue-stained SDS-PAGE (Figure 3C). SEC analysis of freshly purified CysHvCC (0.4 g/L) revealed that, like CysHv, the mutant is purified as a monomer (Figure 3D). Functional characterization showed that the mutant retained inhibitory activity against papain, although with reduced potency compared to the wild-type protein, with 200 nM CysHvCC inhibiting approximately 80% of the activity of 10 nM papain (Figure 3E).

To assess its oligomerization behavior, CysHvCC was subjected to thermal stress at 37 °C, a condition that promotes dimerization of the wild-type protein. SEC analysis over time showed no detectable formation of dimeric species. Instead, incubation at 37 °C, but not at 0 °C, led to the appearance of a minor population of higher-order oligomers (Figure 3F).

The inhibitory activity of CysHvCC after incubation at 37 °C for 48 h showed a decrease in potency, resulting in an increase in residual papain activity from 15% (85% inhibition) for the freshly purified protein to approximately 30% (70% inhibition) (Figure 3G). This decrease occurred in the absence of detectable dimer formation, indicating that loss of activity in the mutant is not associated with the same oligomerization pathway observed for the wild-type protein.

Consistently, the comparative analysis of the electrophoretic profiles of CysHvCC incubated at 37 °C for 0 h and 48 h under both reducing and non-reducing conditions showed no evidence of higher-molecular-mass species or disulfide-mediated oligomers (Figure 3H), confirming that the engineered double mutant remains stable without forming artifactual covalent aggregates over time.

### 2.4. CysHv Dimerization Is Strongly Modulated by pH and Temperature and Correlates with Loss of Inhibitory Activity

During initial expression/purification tests, we observed that the CysHv produced using non-buffered medium, in which the culture reached pH 3, was predominantly recovered as a dimer (Figure S4), suggesting that environmental pH influences its oligomeric state. To explore this possibility, as no high-resolution structure is yet available for CysHv, potential protonation-sensitive features were examined within the predicted structural model.

**Figure 4:**
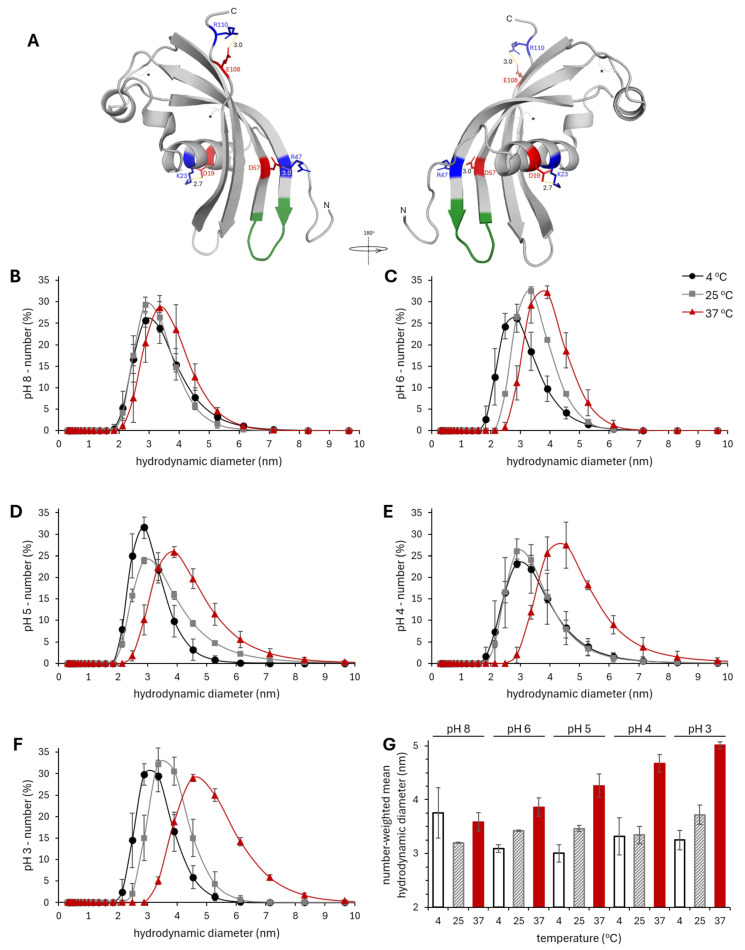
CysHv presents three pairs of oppositely charged residues in close spatial proximity, including one near the L1 loop region, and a temperature- and pH-dependent increase in hydrodynamic diameter. (**A**) Structural model of CysHv highlighting three pairs of oppositely charged residues in close spatial proximity. Positive and negative residues (blue and red, respectively) are shown as sticks, and distances between oppositely charged side-chain atoms are indicated in angstroms. The L1 loop is shown in green. Asterisks (*) indicate disulfide bridges. (**B**–**F**) Aliquots of freshly purified CysHv (pH 8) were buffer-exchanged into sodium citrate buffers at different pH values (6 to 3), as indicated. Samples (0.4 g/L) were incubated at 4 °C (black circles), 25 °C (gray squares), or 37 °C (red triangles) for 72 h and then analyzed by dynamic light scattering (DLS). The graphs show the number (%) as a function of size distribution (nm). (**G**) Number-weighted mean hydrodynamic diameter (nm) of the analyzed samples as a function of temperature and pH. Except in (**A**), data are presented as the mean ± SD of three independent experiments.

Inspection of the CysHv monomer structural model revealed three pairs of oppositely charged residues in close spatial proximity: D19-K23 (α-helix), E108-R110 (C-terminal region), and R47-D57, located in the β-strands flanking the L1 loop (Figure 4A).

PropKa analysis indicated that all of these pairs are consistent with hydrogen-bond interactions (Table S1). The positioning of the R47-D57 pair suggests a possible role in stabilizing the L1-flanking region, hypothetically acting as a pH-dependent electrostatic latch that could contribute to preventing loop extension.

To evaluate the combined effects of pH and temperature on CysHv dimerization, freshly purified protein was buffer-exchanged into sodium citrate buffers ranging from pH 6 to 3, incubated (0.4 g/L) at 4 °C, 25 °C, or 37 °C, and size distribution was assessed by dynamic light scattering (DLS). The analysis after 72 h incubation revealed an increase in hydrodynamic diameter as a function of decreasing pH and increasing temperature (Figure 4B–F). The number-weighted mean hydrodynamic diameter increased from the initial (3.75 ± 0.46) nm under pH 8 at 4 °C, to (5.01 ± 0.06) nm under pH 3 at 37 °C (Figure 4G). These data are consistent with the formation of higher-order species and in agreement with the hydrodynamic diameter prediction derived from Stokes radii estimated by US-SOMO using CysHv structural models.

**Figure 5:**
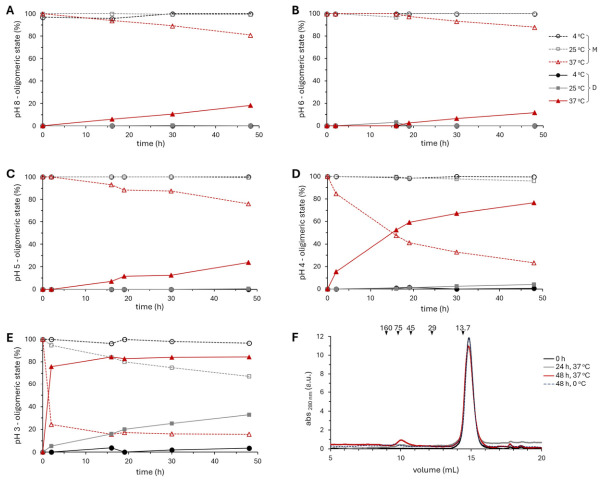
CysHv, but not CysHvCC, presents pH-dependence on temperature-induced dimerization. (**A**–**E**) Freshly purified CysHv (pH 8) was buffer-exchanged to sodium citrate (pH 6–3), as indicated, and incubated (at 0.4 g/L) at 4 °C (black circles), 25 °C (gray squares), or 37 °C (red triangles), and analyzed by SEC. Monomer (M, open symbols and dashed lines) and dimer (D, closed symbols and continuous lines) content (%) over time at indicated pHs are indicated. (**F**) Aliquots of freshly purified CysHvCC (pH 8) were submitted to buffer exchange to sodium citrate buffer (pH 3). Samples (0.4 g/L) were immediately analyzed (0 h) or incubated for 24 h and for 48 h at 37 °C, or 48 h at 0 °C, and analyzed by SEC, as indicated. The graph shows the absorbance (abs) at 280 nm, in arbitrary units (a.u.), as a function of the elution volume. On top, the retention volumes of standard proteins are indicated in kDa. Data are presented as representative results of three independent experiments.

To quantify oligomerization, samples were analyzed by SEC. Because low pH conditions resulted in altered elution behavior, possibly due to column interactions (Figure S5), all samples were analyzed under standardized conditions at pH 8 following the incubation at specific pHs. Control experiments confirmed that oligomeric state distributions were preserved upon buffer exchange (Figure S5), allowing a reliable comparison across conditions. SEC-based analysis confirmed that dimer formation is strongly influenced by both temperature and pH (Figure 5 and Figure S6).

While increased temperature promoted dimer accumulation over time, acidic conditions markedly accelerated this process. At 37 °C, samples at pH 3 reached maximal dimer content (~85%) rapidly, whereas pH 4 required longer incubation but reached similar levels (Figure 5D,E).

In contrast, and further supporting the involvement of structural rearrangement for CysHv dimerization, the conformationally constrained mutant CysHvCC did not form detectable dimeric species even at pH 3 under thermal stress, although the presence of a minor population of higher-order species is still observed at 37 °C in acidic incubation (Figure 5F).

Due to the presence of a predicted free cysteine residue, in order to rule out the involvement of intermolecular disulfide-mediated dimers in our observations, the electrophoretic profile of samples submitted to thermal stress at different pH values was evaluated under non-reducing conditions (Figure S7). After treatment for 72 h at 37 °C, CysHv migrated predominantly as a monomer across all pH conditions tested (pH 8 to 3), further suggesting that the structural rearrangements observed under these conditions do not involve covalent stabilization (Figure S7A). Additional minor, discrete bands were observed exclusively at pH 8, in an amount that does not correlate with the dimerization observed by SEC (Figure 2B), which are likely associated with artifacts during sample preparation. Visually, the electrophoretic profile displays an absence of prominent intermolecularly disulfide-linked populations under the oligomerization parameters. Similar to the wild-type protein, as expected, a predominantly monomeric profile was also observed under non-reducing conditions for the CysHvCC mutant across the tested pH values (Figure S7B).

**Figure 6:**
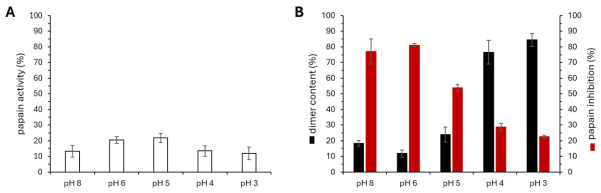
CysHv temperature- and pH-dependent dimerization is associated with reduced inhibitory activity. (**A**) Freshly purified CysHv samples were buffer-exchanged into the indicated pH conditions and immediately tested as papain inhibitors at 50 nM. (**B**) CysHv samples were incubated at 37 °C for 48 h at the indicated pH values. Dimer content (%) and papain inhibition (%) at 50 nM were then determined. Data are presented as the mean ± SD of three independent experiments.

To evaluate the functional consequences of CysHv dimerization, its inhibitory activity against papain was assessed on samples submitted to thermal stress under the different pH conditions (Figure 6).

Immediately after buffer exchange, CysHv retained inhibitory activity across the tested pH range (Figure 6A), indicating that pH alone does not impair function. However, as expected for a dimerization mechanism mediated by domain swapping in which the inhibitory triad is disrupted, after incubation at 37 °C for 48 h, a progressive reduction in CysHv inhibitory activity was observed, which correlated with the extent of dimer formation (Figure 6B).

A relevant reduction in inhibitory activity was already observed at pH 5 (~25% reduction, compared to pH 8), despite only a modest increase in the dimer content being detected (~5% increase, compared to pH 8) (Figure 6B). This behavior may reflect early conformational changes preceding detectable dimer formation, conformational intermediates not resolved as discrete oligomeric species by SEC, or additional pH-dependent effects on protein stability, which remains to be elucidated.

### 2.5. Charge-Complementary Residues in L1 Loop-Flanking ß-Strands Are Enriched in Extracellular Cystatins and Absent in Intracellular Homologs

**Figure 7:**
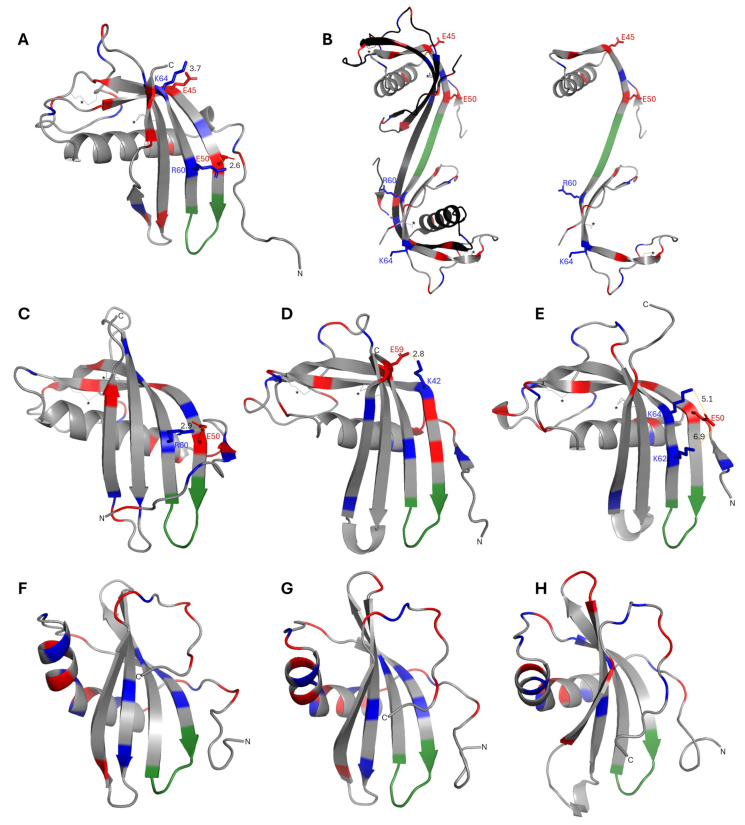
Positioning of charge-complementary residues in L1 loop-flanking β-strands across homologous cystatins from hematophagous organisms. (**A**,**B**) Example of an experimentally determined domain-swapped dimer. Extracellular Sialostatin L was modeled as a monomer (**A**), revealing two pairs of charge-complementary residues in close spatial proximity within the β-strands flanking the L1 loop, which become separated upon domain-swapped dimerization (PDB: 4ZM8) (**B**). The domain-swapped structure is shown with both chains colored black and gray (**left**) and, for clarity, as a single chain (**right**). (**C**–**E**) Examples of extracellular cystatins crystallized as monomers: Sialostatin L2 (PDB: 3LH4) (**C**), Om-cystatin 2 (PDB: 3L0R) (**D**), and Mialostatin (PDB: 6ztk) (**E**). These structures reveal charge-complementary residues in L1 loop-flanking β-strands; however, in Mialostatin, these residues are not in close spatial proximity. (**F**–**H**) Examples of intracellular cystatins: RHcyst-1 (**F**), Bmcystatin (**G**), and TtcysB (**H**). Modeled structures revealed the absence of charge-complementary residues in L1 loop-flanking β-strands. All structures are shown in cartoon representation, with disulfide bonds indicated as lines (indicated by *) and the inhibitory L1 loop highlighted in green. The N- and C-termini are indicated by N and C, respectively. Positively and negatively charged residues are shown in blue and red, respectively. Charge-complementary residues in L1 loop-flanking β-strands are shown as sticks, and distances between oppositely charged side-chain atoms are indicated in angstroms.

To explore whether the presence and spatial arrangement of oppositely charged residues observed in the CysHv model structure (Figure 4A) could extend to other cystatins, a comparative structural analysis was performed using experimentally determined and modeled structures from hematophagous organisms (Figure 7 and Figures S8–S10).

As a reference, the secreted cystatin Sialostatin L [29], for which a domain-swapped dimer (PDB: 4zm8) has been experimentally described [33], was analyzed. Modeling of its monomeric form revealed two pairs of oppositely charged residues in close spatial proximity within the β-strands flanking the L1 loop (Figure 7A). In the domain-swapped dimer, these interactions are disrupted due to the conformational rearrangement in which the L1 loop is extended and adopts a β-strand-like conformation (Figure 7B). A similar pattern was observed by the analysis of Amacstatin 2, a tick cystatin yet to be characterized, that crystallized as a domain-swapped dimer (PDB: 8r29), and even for human cystatin C (hCC), a classic example of a cystatin domain-swapped dimer [23], for which a disulfide bridge-stabilized monomer structure [54] is also available (Figure S8).

Analysis of additional extracellular cystatins, Sialostatin L2 [33], Om-cystatin 2 [31,34], Mialostatin [42] (Figure 7), Om-cystatin 1 [31]; HlSC-1 [27]; RHcyst-2 [36]; HcCyst-3 [38], DsCystatin [39], Iristatin [41], Ricistatin [44], and Amacstatin 1 [56] (Figure S9) revealed that the presence of oppositely charged residues in L1 loop-flanking β-strands as observed for CysHv (Figure 4A) is a recurrent feature (Figure 7C–E and Figure S9). A notable exception is Amblyostatin 1 [60], which lacks oppositely charged residues in this region (Figure S9). In several cases, these residues are positioned at distances compatible with interaction (Figure 7C,D). In contrast, in the analyzed cystatins described to be associated with digestive environments, Mialostatin [42] (Figure 7E), and Om-cystatin 1 [31] and HcCyst-3 [38] (Supplementary Figure S9G–H), although similarly positioned charged residues can be identified, they are separated by larger distances and are not predicted to engage in stabilizing interactions, according to PropKa analysis (Table S1). PropKa results indicated that, in cases where residues were in close spatial proximity, their interactions are consistent with protonation-sensitive sidechain hydrogen bond formation accompanied by significant pKa shifts in the involved residues (Table S1).

In contrast, analysis of modeled structures of intracellular cystatins Ttcysb [28], RHcyst-1 [37], Bmcystatin [30] (Figure 7), and HcCys1 [27], Sjcystatin [35] and PnCystB [43] (Figure S10), revealed a consistent absence of oppositely charged residues in the β-strands flanking the L1 loop (Figure 7F–H and Figure S10), suggesting that this structural feature is not a general property of the cystatin fold but may be associated with secreted salivary proteins in hematophagous.

Analysis of a stefin (Em-Cystatin B) from a non-hematophagous tissue parasite, recently characterized as a tetramer with unknown biological function, and composed of two domain-swapped dimers linked by a disulfide bridge [58], revealed that the monomer presents the same charge-complementarity in the β-strands (Supplementary Figure S8E). This finding reinforces the association between the feature and domain-swapping dimerization.

## 3. Discussion

In a search for new biomolecules with potential biotechnological applications, CysHv, a transcript coding for a secreted cystatin, was identified in the transcriptomic analysis of salivary complexes of the Brazilian leech *Haementeria vizzotoi* [11], and the corresponding protein was previously described as a potent inhibitor of papain and human cathepsin L cysteine proteases [12].

Cystatins derived from hematophagous organisms, primarily by targeting host cysteine proteases involved in pathological pathways, are widely recognized as potential frameworks for developing novel immunomodulatory, anti-inflammatory, antiviral, or antitumor agents [29,34,35,36,37,38,39,40,41,42,43,44,45,61,62]. Importantly, however, successfully and rationally deploying these molecules in complex functional or cell-based assays requires a thorough understanding of their structural stability under physiological conditions. Since structural changes, domain-swapping dimerization, for instance, can drastically alter functional availability, establishing how temperature and pH modulate the protein stability and inhibitory activity represents a mandatory baseline requirement prior to any biological evaluation.

To address this, the present study extends the characterization of CysHv by demonstrating that its inhibitory function is environment-dependent and closely linked to its oligomeric state.

Biophysical analysis showed that purified CysHv adopts a well-folded structure, with secondary structure content consistent with a canonical cystatin fold. Its structural integrity and fold were experimentally validated in solution using far-UV CD spectroscopy. While the proportions estimated by CD spectrum deconvolution for α-helices and turns closely mirrored those assigned by the DSSP algorithm from the predicted 3D coordinates, modeled with Robetta, a slight quantitative discrepancy was observed in β-strand content (39.8% experimental versus 49.1% predicted). This variation is likely attributable to the inherent limitations of static computational models when compared to the dynamic conformational flexibility of proteins in solution. Additionally, this divergence was further influenced by the restricted wavelength range used for deconvolution (200–250 nm). This narrow window was required to avoid the high background noise below 200 nm caused by the NaCl-containing buffer, which limits the extraction of far-UV spectral features critical for precise β-sheet estimation. Nevertheless, this experimental characterization provides a robust qualitative agreement with the computational data, successfully confirming the overall reliability of the predicted 3D model and demonstrating that its structural content is fully consistent with the predominantly α + β fold characteristic of the cystatin superfamily.

Despite presenting an unpaired cysteine residue, CysHv is purified as an active monomer. However, stability tests revealed a tendency to form dimers under thermal stress, preventing the reliable investigation of its biological effects on relevant cellular models while motivating further investigation of its oligomeric behavior. Dimerization, which was not associated with intramolecular disulfide-bridge formation in any of the tested conditions, was found to be dependent on time, temperature, and protein concentration, and correlated with loss of inhibitory activity; while monomeric species remained active, dimers were found to be inactive.

Given that domain swapping has been reported for other cystatins [23,33,57,58,59], it was hypothesized that CysHv dimerization could involve a similar mechanism. In this model, the monomer-dimer transition likely involves conformational rearrangements that permit structural exchange between protomers, disrupting the canonical inhibitory interface, and providing a basis for the observed loss of CysHv activity in the dimeric form.

The observed concentration, temperature, and time dependence of CysHv dimerization supports a kinetically controlled process, with an energetic barrier between monomeric and dimeric states. Concentration dependence further suggests that dimer formation requires intermolecular association following access to a conformationally permissive state, while elevated temperature and time increase the likelihood of forming exchanged species [63,64,65,66], consistent with experimental observations on domain-swapping-mediated oligomerization.

To experimentally assess this hypothesis, site-directed mutagenesis was used to introduce an artificial disulfide bond in the CysHv L1-flanking region to constrain the flexibility required for dimerization, adopting a strategy previously explored to stabilize a cystatin scaffold [67]. The resulting double mutant, CysHvCC, was expressed and purified as a monomer and retained inhibitory activity against papain, albeit with reduced potency compared to the wild-type protein. This reduction suggests that structural flexibility in the constrained region may contribute to optimal protease binding. A similar strategy has also been reported for hCC, in which disulfide bond stabilization resulted in a monomeric form with preserved inhibitory activity. In that case, the engineered disulfide bond was introduced in β-strands corresponding to those targeted in CysHvCC, but positioned further from the L1 loop [54]. This difference suggests that the precise positioning of the covalent constraint may influence the balance between stability and inhibitory function. In CysHvCC, the proximity of the engineered disulfide bond to the L1-flanking region may impose additional restrictions that partially impair activity. Ongoing work aims to evaluate alternative disulfide placements to optimize stabilization while preserving potency.

Importantly, the introduction of the covalent constraint adjacent to the L1 loop prevented detectable dimer formation under the tested conditions. While CysHvCC was obtained as a well-structured protein according to CD analysis, potential effects on overall conformation cannot be excluded. However, the specific suppression of dimerization supports the requirement of local flexibility for the dimerization observed in the wild-type protein, and strongly supports the hypothesis that CysHv dimerization involves a conformational transition consistent with a domain-swapping-like mechanism. Additionally, since dimerization under thermal stress prevented reliable investigations of CysHv in cellular models, CysHvCC will be used as a stable version to explore biological and biotechnological applications in future studies.

Although no high-resolution structure of CysHv is available, multiple independent observations converge to support a defined structural rearrangement compatible with a domain-swapping-like mechanism. These include the loss of inhibitory activity upon dimer formation, the agreement between experimentally determined and model-predicted hydrodynamic dimensions, the dependence on concentration, time, and temperature, and the behavior of the disulfide-engineered mutant. Interestingly, under thermal stress, the mutant exhibited the formation of minor higher-order oligomeric species in the absence of detectable dimerization. This observation may suggest that alternative oligomerization pathways become accessible when the conformational route leading to dimer formation is restricted. Supporting that notion, certain hinge loop mutants of hCC, for instance, can form putative hexameric assemblies, without relevant dimer formation [68].

CysHv oligomerization occurred under non-acidic conditions but was strongly modulated by pH, indicating that pH affects the kinetics rather than the equilibrium of dimer formation. While the final oligomeric distribution reached similar levels under acidic conditions (75–85% dimer after 48 h at 37 °C, at pH 3 and 4), decreasing pH markedly accelerated the kinetics of dimer formation. The progressive accumulation of dimer correlated with CysHv loss of inhibitory activity, establishing a direct functional consequence of this conformational transition.

Under mildly acidic conditions (pH 5), an apparent disproportionate activity loss relative to dimer content suggests early conformational changes, potentially involving molten globule-like states [55] or subtle perturbations of the inhibitory interface, may precede detectable oligomerization. However, the molecular basis of this effect remains to be determined.

Together, the concentration-, temperature-, and pH-dependent dimerization of CysHv, which is impaired by the covalent constraint marginal to the L1 loop in the CysHvCC mutant, strongly supports a mechanistic interpretation consistent with domain-swapping-like dimerization and demonstrates that this phenomenon is strongly influenced by environmental conditions and is associated with loss of inhibitory activity, indicating a model in which oligomerization acts as a condition-dependent regulator of function.

Structural analysis of the CysHv monomer model revealed the presence of oppositely charged residues in the β-strands flanking the L1 loop, positioned in close spatial proximity and potentially capable of interaction in a pH-sensitive manner. This observation raised the possibility that such residues may contribute to local stabilization of the monomeric state in a protonation-dependent context, consistent with observations reported for hCC [50,69,70].

To assess whether this feature extends beyond CysHv in hematophagous organisms, a structural analysis was performed using a representative, non-comprehensive set of previously characterized cystatins, including both secreted cystatins and intracellular stefins. Comparative analysis showed that such charged-complementary arrangements are recurrently observed among monomeric structures of secreted salivary cystatins, often at distances compatible with interaction, whereas absent in intracellular cystatins. Similar arrangements are also observed in cystatins known to undergo domain swapping, including the Sialostatin L [33], Amacstatin 2 [57], and human cystatin C [23].

Causality cannot be established, and additional factors are certainly involved to drive domain-swapping dimerization. For hCC, for instance, it is reported that hydrophobic interactions and additional electrostatic interactions are relevant for monomer stabilization [50,69]. However, the recurrent observation of oppositely charged residues in the L1-flanking β-strands of secreted cystatins from hematophagous, suggests that this feature could act as pH-sensitive modulators of local structure, influencing access to conformational states required for dimerization. Rather than directly driving oligomerization, these residues may influence access to conformational states by weakening local electrostatic interactions in a protonation-dependent manner.

Interestingly, some extracellular cystatins described to be associated with digestive environments, where they might need to be active (thus, monomeric) at acidic conditions, such as Mialostatin [42], HcCyst-3 [38], and Om-cystatin 1 [31], lack these close-proximity charged residues.

Amblyostatin 1, a recently described highly basic (predicted pI ~10.6) salivary cystatin [60], derived from a comparative transcriptome analysis of fed and partially unfed *Amblyomma sculptum* ticks [71], represents a clear exception within the analyzed dataset, as it lacks oppositely charged residues in this region, further highlighting its atypical sequence features relative to other cystatins.

Despite this exception, the overall distribution observed across the analyzed dataset supports the proposed interpretation and is consistent with the experimentally observed pH-dependent behavior of CysHv, in which acidic conditions accelerate the conformational transition associated with loss of activity through a domain-swapping-like mechanism.

In this context, protonation of ionizable residues may disrupt stabilizing electrostatic interactions, as demonstrated for human cystatin C, where low pH induces protonation associated with the disruption of interactions within the native structure. Across the analyzed cystatins, such effects may contribute to weakening local interactions in the L1-flanking region, potentially facilitating conformational rearrangements and access to conformationally permissive states under thermal stress.

Within the analyzed proteins from hematophagous species, this structural pattern is consistently observed among secreted cystatins, including those reported to undergo domain swapping, but is absent in intracellular stefins. Given that extracellular proteins are more likely to encounter variable environmental conditions, including fluctuations in pH, the presence of such a feature may be consistent with an adaptation to modulate conformational behavior in response to external cues. Conversely, the absence of this arrangement in intracellular cystatins, which typically operate under more tightly regulated physicochemical conditions, suggests that such pH-sensitive modulation may not be required in these environments.

Some methodological limitations regarding the structural analysis must be addressed. Although the computational models provided a valuable framework to investigate potential protonation-sensitive features, these atomic coordinates remain predictive. In the absence of high-resolution structural data for CysHv and the other modeled superfamily members, as well as evaluations by molecular dynamics, the exact spatial arrangement of the R47-D57 pair and analogs and their role as electrostatic latches must be interpreted as a working hypothesis. Nevertheless, this hypothesis is strongly supported by our findings that a similar electrostatic arrangement is consistently shared among other secreted cystatins, including those with experimentally solved structures (PDB codes 3LH4, 3L0R, 8R28, and 5O46), while being absent in intracellular ones. This structural conservation provides a robust biological rationale for our models, although future high-resolution structural studies will be essential to experimentally confirm the precise orientation of these conserved residue pairs in CysHv and other modeled superfamily members.

While this interpretation is still hypothetical, it is consistent with the observed distribution of oppositely charged residues and supports the idea that local structural determinants may be linked to protein localization and functional context.

Furthermore, the presence of the discussed structural pattern among secreted cystatins could point to a tendency toward protein dimerization under specific environmental conditions, potentially in a domain-swapping-like fashion. This possibility might justify further case-to-case investigations of these proteins.

From a functional perspective, the condition-dependent inactivation of CysHv raises the possibility that oligomerization may act as a regulatory mechanism. In hematophagous organisms, cystatins are secreted in saliva to modulate host proteases during feeding. It is tempting to speculate that environmental transitions, such as pH changes upon re-ingestion, could promote loss of inhibitory activity via domain-swapping-mediated dimerization, potentially preserving digestive protease function. Although this model requires further validation, it highlights a possible link between environmental sensing and functional regulation.

Domain swapping dimerization of cystatins from hematophagous organisms has so far been reported primarily in crystallographic contexts [33,57] and, to our knowledge, has not been previously characterized in solution. The present results suggest that this mechanism may be more broadly accessible within the cystatin superfamily than previously appreciated.

A recent study further supports this notion by describing an intracellular cystatin from a tissue parasite, EmCystatin-B, undergoing domain-swapping dimerization associated with disulfide-mediated tetramerization [58]. Structural modeling of its monomeric state revealed features consistent with those identified here, suggesting that similar determinants may operate across different cystatin families under appropriate conditions.

Taken together, these findings emphasize the importance of evaluating recombinant proteins across environmentally relevant conditions. Protein function is often characterized under a limited set of experimental conditions, which may obscure conformational transitions with significant functional consequences. In this context, dimer-prone CysHv provides a clear example of how environmental parameters such as pH and temperature can modulate both structure and function, reinforcing the need for broader experimental exploration. This is particularly relevant for hematophagous cystatins, whose functional properties make them attractive candidates for biotechnological applications in several environmental contexts.

## 4. Conclusions

This study demonstrates that the inhibitory activity of CysHv is closely linked to its conformational state, with dimerization leading to functional inactivation. The protein undergoes a time-, temperature-, and concentration-dependent monomer-dimer transition, which is accelerated under acidic conditions but not strictly dependent on them. Covalent restriction of the L1-flanking region prevented dimer formation, supporting a requirement for local conformational flexibility in this transition. The combined biochemical, biophysical, and mutational evidence points to a defined oligomerization pathway compatible with a domain-swapping-like mechanism. Complementary structural analysis suggests that local electrostatic features in the L1-flanking region may contribute to modulating access to conformationally permissive states in a pH-dependent manner. Although this aspect remains speculative, its recurrence among related cystatins highlights a potential structural determinant linking environmental conditions to conformational behavior. Overall, CysHv illustrates how environmental factors can regulate both structure and function, reinforcing the importance of evaluating protein dynamics beyond standard conditions and highlighting the broader relevance of condition-dependent transitions within the cystatin superfamily.

## 5. Materials and Methods

### 5.1. CysHv-Expressing Pichia pastoris Clone

*Pichia pastoris* (currently known as *Komagataella phaffii*) X-33 was genetically modified by genomic integration of the pD912-AK expression vector (ATUM 2.0, Newark, NJ, USA) carrying a synthetic codon-optimized sequence encoding CysHv (pD912-AK/340), as previously described [13].

### 5.2. CysHvCC-Expressing Pichia pastoris Clone

The CysHv expression plasmid pD912-AK/340 was subjected to site-directed mutagenesis to generate pD912-AK/CysHvCC, encoding the double cysteine mutant Q49C and N55C. Mutagenesis was performed by PCR using the Phusion™ Site-Directed Mutagenesis Kit (Thermo Scientific, Waltham, MA, USA), with primers designed to introduce the desired substitutions (mutated nucleotides underlined):

Forward: 5′-TGCGTGGTTGCTGGAATGTGCTACGATTTGACAGT-3′ and

Reverse: 5′-GTTACGAGCAGAAATCACCCTTTGGTTACG-3′.

These primers (25 μM each) were initially phosphorylated in a reaction containing 1 U T4 polynucleotide kinase, 0.5 mM ATP, and 1 X T4 polynucleotide kinase buffer (all from New England Biolabs—NEB, Ipswich, MA, USA), performed at 37 °C for 30 min. Polynucleotide kinase was then inactivated at 70 °C for 10 min, and the DNA was purified using the QIAquick PCR Purification Kit (Qiagen, Hilden, Germany). For the mutagenic PCR, template pD912-AK/340 (10 ng), phosphorylated primers forward and reverse (5 μM each), dNTP mix (0.2 mM), Phusion™ II hot start DNA polymerase (1 U), and Phusion™ HF buffer (1 X) were mixed and used in a 2-step reaction. The cycling conditions were as follows: for denaturing, 98 °C, 30 s; for annealing/extension, 30 cycles at 96 °C, 10 s, and 72 °C, 4 min; followed by a final extension at 72 °C for 5 min. After amplification, the parental plasmid was digested with FastDigest *Dpn*I (Thermo Scientific) for 15 min at 37 °C, and the mutated plasmid was subjected to a ligation reaction performed using T4 DNA ligase (Thermo Scientific). The resulting product was transformed into *Escherichia coli* DH5α cells. Transformants were selected on LB-low salt agar (10 g/L tryptone, 5 g/L yeast extract, 5 g/L NaCl, and 15 g/L agar) plates containing 100 μg/mL zeocin. Plasmid DNA was isolated from selected zeocin-resistant *E. coli* DH5α clones for analysis. The presence of the intended mutations was confirmed by nucleotide sequencing. The confirmed plasmid pD912-AK/CysHvCC was then linearized with *Sac*I and electroporated into competent *Pichia pastoris* X-33 cells. Transformants were selected on YPD agar plates supplemented with 100 μg/mL zeocin, and genomic integration of the CysHvCC construct was verified by colony PCR, using both the parental plasmid (pD912-AK/CysHvCC) and *P. pastoris* X33-CysHv clone (designed as WT) as positive controls, with primers specifically designed to amplify the CysHv coding sequence (forward: 5′-AAAGGTTTACTTGGTGGTTGG-3′ and reverse: 5′-AATGGATCTAAATTCGCCGCAGCT-3′). Several positive clones were identified and subjected to small-scale expression screening to select the best-producing transformant. Selected clones were stored as glycerol stocks (14% *v*/*v*) at −80 °C. Oligonucleotides were synthesized by EXXTEND (Paulínia, SP, Brazil).

### 5.3. CysHv and CysHvCC Expression and Purification

Expression of CysHv and CysHvCC was performed following the same protocol. A single colony from freshly plated glycerol stocks was used to inoculate 25 mL of BMGY medium (100 mM potassium phosphate, pH 6.0, 1% yeast extract, 2% peptone, 1.34% YNB, 4 × 10^−5^% D-biotin, and 1% glycerol). Cultures were grown for approximately 18 h at 30 °C and 250 rpm and used to inoculate 500 mL of fresh BMGY medium at 2.5% (*v*/*v*). Cells were cultured at 30 °C and 250 rpm until the optical density at 600 nm (OD_600_) reached approximately 2.0. Cultures were then centrifuged (1500× *g*, 15 min, 4 °C), and cell pellets were resuspended in BMMY medium (100 mM potassium phosphate, pH 6.0, 1% yeast extract, 2% peptone, 1.34% YNB, 4 × 10^−5^% D-biotin, and 0.5% methanol) to an OD_600_ of 1.0. Protein expression was induced for 48 h at 30 °C and 250 rpm, with methanol supplementation to a final concentration of 0.5% (*v*/*v*) after 24 h. After induction, cultures were centrifuged (3500× *g*, 15 min, 4 °C), and the supernatant containing the secreted recombinant protein was collected and filtered through a 0.22 μm membrane. Clarified supernatant was adjusted for hydrophobic interaction chromatography (HIC) by the addition of solid ammonium sulfate to a final concentration of 1 M (144 g/L). HIC was performed using a 5 mL HiTrap Phenyl FF High Sub (HS) column (Cytiva, Uppsala, Sweden) connected to an ÄKTA Avant system (Cytiva), with approximately 500 mL of supernatant loaded per run. The column was equilibrated with buffer A (1 M ammonium sulfate, 20 mM Tris-HCl, pH 8.0, 50 mM NaCl, 1 mM EDTA). After sample loading, the column was washed with 20 column volumes (CV) of buffer A, and proteins were eluted using a linear gradient from 0 to 100% buffer B (20 mM Tris-HCl, pH 8.0, 50 mM NaCl, 1 mM EDTA). Fractions were analyzed by 15% SDS-PAGE followed by Coomassie blue staining, and those containing the target protein were pooled. Pooled fractions were desalted using a HiPrep 26/10 desalting column (Cytiva) equilibrated with 20 mM Tris-HCl, pH 8.0, and 1 mM EDTA. Samples were processed in multiple sequential runs (10 mL per injection). Desalted material was further purified by ion exchange chromatography (IEX) using a 5 mL HiTrap Q FF column (Cytiva), equilibrated with buffer A (20 mM Tris-HCl, pH 8.0, 1 mM EDTA). After injection, the column was washed with 10 c.v. Buffer A and proteins were eluted with a linear gradient from 0 to 30% buffer B (20 mM Tris-HCl, pH 8.0, 1 mM EDTA, 1 M NaCl). Fractions were analyzed by 15% SDS-PAGE and Coomassie blue staining, and those containing the target protein were pooled. Pooled fractions were concentrated using Amicon ultrafiltration devices (3 kDa molecular mass cutoff) and subjected to size exclusion chromatography on a Superdex 75 16/600 column (Cytiva) equilibrated with buffer (20 mM Tris-HCl, pH 8.0, 50 mM NaCl, 1 mM EDTA). Fractions were analyzed by 15% SDS-PAGE followed by Coomassie blue staining, and those containing the purified protein were pooled and used for subsequent assays. The typical protein concentration after purification was approximately 0.4 g/L, as determined by UV absorbance (280 nm) using a BioDrop spectrophotometer (Biochrom, Cambridge, UK), applying the Warburg–Christian correction.

### 5.4. Circular Dichroism (CD) Spectroscopy

CD spectra were recorded on a JASCO J-810 spectropolarimeter (JASCO, Tokyo, Japan) equipped with a thermoelectric temperature controller (Peltier system), with the sample temperature maintained at 20 °C, using Spectra Manager software (version 1.53.04). CysHv (~10 µM) was analyzed in buffer at pH 8.0 (20 mM Tris-HCl, 50 mM NaCl, 1 mM EDTA). A buffer-only sample was used for baseline correction. Far-UV CD spectra were collected in the range of 200–260 nm, with seven accumulations, using a quartz cuvette with a 1.0 mm pathlength. Spectra were recorded in millidegrees (mdeg), baseline-corrected by buffer subtraction, and converted to mean residue ellipticity (MRE), expressed in deg·cm^2^·dmol^−1^. The experimental CD spectrum of CysHv was deconvoluted for secondary structure estimation using the BeStSel server (https://bestsel.elte.hu/ (accessed on 15 April 2024)) within the 200–250 nm wavelength range.

### 5.5. Dynamic Light Scattering (DLS)

Samples (100 μL) subjected to different treatments (namely, thermal treatment at 4 °C, 25 °C, and 37 °C at pH 3, 4, 5, 6, and 8, for up to 72 h) were analyzed at 20 °C, after 2 min equilibrium, using ZEN0040 cuvettes in a Zetasizer Pro Red instrument (Malvern Panalytical, Malvern, UK), using the software ZS Xplorer (version 3.0.0.53). Scattering was measured at a detection angle of 173°, using a laser wavelength of 632.8 nm.

### 5.6. Oligomeric State pH-Dependence Analyzed by Size Exclusion Chromatography (SEC)

Protein oligomeric state was assessed by size exclusion chromatography (SEC) using a Superdex 75 10/300 column (23.562 mL column volume; Cytiva), with a 100 μL injection volume and a flow rate of 0.5 mL/min, at room temperature (~20 °C). For analysis of freshly purified samples, the column was equilibrated with purification buffer (20 mM Tris-HCl, pH 8.0, 50 mM NaCl, 1 mM EDTA). To evaluate the effect of pH on protein oligomeric state, samples purified at pH 8.0 were first concentrated to ~2 g/L using Amicon ultrafiltration devices (Millipore, Burlington, MA, USA) (3 kDa cutoff) and subjected to buffer exchange using a 5 mL HiTrap desalting column (Cytiva) equilibrated with buffers at the desired pH values. After buffer exchange, samples were adjusted to a final concentration of 0.4 g/L. As a control, samples maintained at pH 8.0 were subjected to the same procedure.

Buffers covering the pH range 3.0–6.0 were prepared using 20 mM sodium citrate, with pH adjusted by appropriate proportions of citric acid and sodium citrate. All buffers contained 50 mM NaCl. Due to nonspecific interactions between the protein and the column matrix at lower pH values, all SEC runs were performed under identical chromatographic conditions using a pH 8.0 buffer (20 mM Tris-HCl, pH 8.0, 50 mM NaCl, 1 mM EDTA), regardless of the sample buffer. Therefore, the observed oligomeric distributions reflect the conformational state of the protein established prior to injection. Commercial globular protein standards (Cytiva) were used for column calibration and estimation of apparent molecular masses.

### 5.7. Papain Activity Assay

Papain was used as a model cysteine protease. Assays were performed as previously described [72], with modifications. The enzyme was pre-activated for 15 min on ice in activation buffer containing 50 mM sodium phosphate, pH 6.0, 200 mM NaCl, 5 mM EDTA, and 6 mM DTT. Enzymatic activity (10 nM papain) was measured in the presence of purified cystatins (0, 5, 10, 25, 50, or 100 nM for CysHv, and 0, 50, 100, 200, or 500 nM for CysHvCC), using 200 μM of the fluorogenic substrate Z-FR-AMC (Sigma-Aldrich, St. Louis, MO, USA). Reactions were carried out at 30 °C for 10 min in activity buffer (activation buffer without DTT), in a final volume of 100 μL, in 96-well plates. Fluorescence associated with AMC release was monitored using a SpectraMax M5 multimode microplate reader (Molecular Devices, San Jose, CA, USA) (λ excitation = 380 nm, λ emission = 480 nm). Protein samples were diluted in their respective buffers to allow the addition of 5 μL per reaction. Control reactions containing 5 μL of the corresponding buffers without protein were included and considered as zero inhibitor concentration. No significant effects on papain activity were observed upon buffer addition compared to reactions performed in activity buffer alone. Initial velocities, expressed in arbitrary units of fluorescence (a.u.f.) per minute, were calculated from the slope of the linear regression line of product formation (a.u.f. versus time) restricted to the first 10 min of the reaction, and used to quantify enzyme activity.

### 5.8. In Silico Analysis

CysHv and CysHvCC were modeled as a monomer using the Robetta server (https://robetta.bakerlab.org/submit.php (accessed on 17 April 2024)), applying the deep learning-based RoseTTAFold method. The secondary structure composition of the CysHv tridimensional structure model, predicted via the Robetta server, was analyzed using the Dictionary of Protein Secondary Structure (DSSP) algorithm (https://pdb-redo.eu/dssp (accessed on 26 June 2026)). The percentage of each structural element was calculated based on the individual assignment of every amino acid residue within the output file. Secondary structure categories were grouped as follows: β-strand (E, B), α-helix (H), 3_10_-helix (G), turn (T), bend (S), and coils/loops (unassigned residues). A domain-swapped dimer model of CysHv was generated by homology modeling using SWISS-MODEL (https://swissmodel.expasy.org/ (accessed on 18 April 2024)) and the dimeric structure of Sialostatin L (PDB: 4zm8) as a structural scaffold. Hydrodynamic parameters of monomeric and dimeric CysHv were estimated using the US-SOMO web server (https://somoweb.genapp.rocks/somoweb/ (accessed on 15 October 2025)) based on the structural models. Amino acid sequences of additional proteins included in the analysis were retrieved from GenBank or UniProt. The presence of signal peptide was predicted using SignalP 6.0 (https://services.healthtech.dtu.dk/services/SignalP-6.0/ (accessed on 10 March 2026)), and its presence or absence was used to classify the cystatins as extracellular or intracellular, respectively. Experimental structures were retrieved from the Protein Data Bank (PDB; https://www.rcsb.org/ (accessed on 27 March 2026)) using their respective accession codes. When unavailable, structural models were obtained from AlphaFold2 via ColabFold (https://colab.research.google.com/github/sokrypton/ColabFold/blob/main/AlphaFold2.ipynb (accessed on 28 March 2026)) or from the Robetta server (https://robetta.bakerlab.org/ (accessed on 17 April 2024)), as indicated. Structural visualization and interatomic distance measurements were performed using PyMOL (version 3.1.5.1). These computational models reflect structural predictions generated via homology and deep learning-based analyses in the absence of high-resolution experimental coordinates and therefore carry the inherent limitations of in silico modeling. PROPKA analysis (https://www.ddl.unimi.it/vegaol/propka.htm (accessed on 30 March 2026)) was performed using experimental and modeled structures to assess potential electrostatic interactions and estimate residue pKa values. Residues located in the β-strands flanking the L1 loop were inspected for oppositely charged side chains, and predicted interaction energies and classifications were recorded.

## Data Availability

The original contributions presented in this study are included in the article/Supplementary Materials. Further inquiries can be directed to the corresponding author.

## Supplementary Materials

The following supporting information can be downloaded at https://www.mdpi.com/article/10.3390/toxins18070300/s1. Figure S1: Representative results of CysHv expression and purification scheme; Figure S2: Obtention of double mutant CysHvCC.; Figure S3: Representative expression and purification of the CysHv double mutant (CysHvCC); Figure S4: CysHv purified predominantly as a dimer under acidic conditions (pH 3); Figure S5: Oligomeric state distribution of CysHv determined by SEC at pH 3 and pH 8; Figure S6: Representative SEC chromatograms showing time-, temperature-, and pH-dependent changes in the oligomeric distribution of CysHv; Figure S7: Non-reducing electrophoretic profiles of CysHv and CysHvCC submitted to thermal stress under different pH conditions; Figure S8: Additional examples of the spatial arrangement of charge-complementary residues in L1 loop-flanking β-strands across cystatins crystallized as domain-swapped dimers; Figure S9: Additional examples of the spatial arrangement of charge-complementary residues in L1 loop-flanking β-strands across homologous secreted cystatins from hematophagous organisms; Figure S10: Additional intracellular cystatins showing absence of proximal oppositely charged residues in L1 loop-flanking β-strands; Table S1: PropKa-based analysis of charge-complementary residues in L1 loop-flanking β-strands of selected cystatins.

## Abbreviations

The following abbreviations are used in this manuscript:

SEC	Size Exclusion Chromatography
CD	Circular Dichroism
DLS	Dynamic Light Scattering
Tris	Tris(hydroxymethyl)aminomethane
EDTA	Ethylenediaminetetraacetic Acid

## References

[1] Niu C., Zhang J., Okolo P.I. (2026). Plant-Derived Diterpenoids as Potential Chemotherapeutics for Gastric Cancer. Curr. Oncol. Rep..

[2] Xie X., Zhai Y., Cheng H., Wei W.H., Ren M. (2025). From Taxus to paclitaxel: Opportunities and challenges for urban agriculture to promote human health. Plant Physiol. Biochem..

[3] Ferreira S.H., Bartelt D.C., Greene L.J. (1970). Isolation of bradykinin-potentiating peptides from Bothrops jararaca venom. Biochemistry.

[4] Hayashi M.A., Camargo A.C. (2005). The Bradykinin-potentiating peptides from venom gland and brain of Bothrops jararaca contain highly site specific inhibitors of the somatic angiotensin-converting enzyme. Toxicon.

[5] Markwardt F. (1993). Hirudin: The famous anticoagulant agent. Adv. Exp. Med. Biol..

[6] Raghavi S., Deva Darshini B., Saravanan K.M., Anbarasu K. (2026). Molecular Insights into Leech-Derived Bioactive Compounds: Biochemical Mechanisms and Therapeutic Potential. Int. J. Mol. Sci..

[7] Arcà B., Ribeiro J.M. (2018). Saliva of hematophagous insects: A multifaceted toolkit. Curr. Opin. Insect Sci..

[8] Jmel M.A., Voet H., Araújo R.N., Tirloni L., Sá-Nunes A., Kotsyfakis M. (2023). Tick Salivary Kunitz-Type Inhibitors: Targeting Host Hemostasis and Immunity to Mediate Successful Blood Feeding. Int. J. Mol. Sci..

[9] Chmelař J., Kotál J., Langhansová H., Kotsyfakis M. (2017). Protease Inhibitors in Tick Saliva: The Role of Serpins and Cystatins in Tick-host-Pathogen Interaction. Front. Cell. Infect. Microbiol..

[10] Černý J., Arora G. (2024). Proteases and protease inhibitors in saliva of hard ticks: Biological role and pharmacological potential. Adv. Parasitol..

[11] Amorim A.M., de Oliveira U.C., Faria F., Pasqualoto K.F., Junqueira-de-Azevedo Ide L., Chudzinski-Tavassi A.M. (2015). Transcripts involved in hemostasis: Exploring salivary complexes from *Haementeria vizottoi* leeches through transcriptomics, phylogenetic studies and structural features. Toxicon.

[12] Linhares D.D.C., Faria F., Kodama R.T., Amorim A.M.X.P., Portaro F.C.V., Trevisan-Silva D., Ferraz K.F., Chudzinski-Tavassi A.M. (2021). Novel Cysteine Protease Inhibitor Derived from the *Haementeria vizottoi* Leech: Recombinant Expression, Purification, and Characterization. Toxins.

[13] Kordis D., Turk V. (2009). Phylogenomic analysis of the cystatin superfamily in eukaryotes and prokaryotes. BMC Evol. Biol..

[14] Turk V., Stoka V., Turk D. (2008). Cystatins: Biochemical and structural properties, and medical relevance. Front. Biosci..

[15] Shamsi A., Bano B. (2017). Journey of cystatins from being mere thiol protease inhibitors to at heart of many pathological conditions. Int. J. Biol. Macromol..

[16] Alvarez-Fernandez M., Barrett A.J., Gerhartz B., Dando P.M., Ni J., Abrahamson M. (1999). Inhibition of mammalian legumain by some cystatins is due to a novel second reactive site. J. Biol. Chem..

[17] Dall E., Hollerweger J.C., Dahms S.O., Cui H., Häussermann K., Brandstetter H. (2018). Structural and functional analysis of cystatin E reveals enzymologically relevant dimer and amyloid fibril states. J. Biol. Chem..

[18] Tušar L., Usenik A., Turk B., Turk D. (2021). Mechanisms Applied by Protein Inhibitors to Inhibit Cysteine Proteases. Int. J. Mol. Sci..

[19] Gao X., Tian Y., Liu Z.L., Li D., Liu J.J., Yu G.X., Duan D.Y., Peng T., Cheng T.Y., Liu L. (2024). Tick salivary protein Cystatin: Structure, anti-inflammation and molecular mechanism. Ticks Tick Borne Dis..

[20] Barrett A.J. (1986). The cystatins: A diverse superfamily of cysteine peptidase inhibitors. Biomed. Biochim. Acta.

[21] Rawlings N.D., Waller M., Barrett A.J., Bateman A. (2014). MEROPS: The database of proteolytic enzymes, their substrates and inhibitors. Nucleic Acids Res..

[22] Abrahamson M., Alvarez-Fernandez M., Nathanson C.M. (2003). Cystatins. Biochem. Soc. Symp..

[23] Janowski R., Kozak M., Jankowska E., Grzonka Z., Grubb A., Abrahamson M., Jaskolski M. (2001). Human cystatin C, an amyloidogenic protein, dimerizes through three-dimensional domain swapping. Nat. Struct. Biol..

[24] Rawlings N.D., Barrett A.J. (1990). Evolution of proteins of the cystatin superfamily. J. Mol. Evol..

[25] Schwarz A., Valdés J.J., Kotsyfakis M. (2012). The role of cystatins in tick physiology and blood feeding. Ticks Tick Borne Dis..

[26] Martins L.A., Kotál J., Bensaoud C., Chmelař J., Kotsyfakis M. (2020). Small protease inhibitors in tick saliva and salivary glands and their role in tick-host-pathogen interactions. Biochim. Biophys. Acta Proteins Proteom..

[27] Newlands G.F., Skuce P.J., Knox D.P., Smith W.D. (2001). Cloning and expression of cystatin, a potent cysteine protease inhibitor from the gut of *Haemonchus contortus*. Parasitology.

[28] Lefebvre C., Cocquerelle C., Vandenbulcke F., Hot D., Huot L., Lemoine Y., Salzet M. (2004). Transcriptomic analysis in the leech *Theromyzon tessulatum*: Involvement of cystatin B in innate immunity. Biochem. J..

[29] Kotsyfakis M., Sá-Nunes A., Francischetti I.M., Mather T.N., Andersen J.F., Ribeiro J.M. (2006). Antiinflammatory and immunosuppressive activity of sialostatin L, a salivary cystatin from the tick *Ixodes scapularis*. J. Biol. Chem..

[30] Lima C.A., Sasaki S.D., Tanaka A.S. (2006). Bmcystatin, a cysteine proteinase inhibitor characterized from the tick *Boophilus microplus*. Biochem. Biophys. Res. Commun..

[31] Grunclová L., Horn M., Vancová M., Sojka D., Franta Z., Mares M., Kopácek P. (2006). Two secreted cystatins of the soft tick *Ornithodoros moubata*: Differential expression pattern and inhibitory specificity. Biol. Chem..

[32] Yamaji K., Tsuji N., Miyoshi T., Islam M.K., Hatta T., Alim M.A., Anisuzzaman M., Kushibiki S., Fujisaki K. (2009). A salivary cystatin, HlSC-1, from the ixodid tick *Haemaphysalis longicornis* play roles in the blood-feeding processes. Parasitol. Res..

[33] Kotsyfakis M., Horka H., Salat J., Andersen J.F. (2010). The crystal structures of two salivary cystatins from the tick *Ixodes scapularis* and the effect of these inhibitors on the establishment of Borrelia burgdorferi infection in a murine model. Mol. Microbiol..

[34] Salát J., Paesen G.C., Rezácová P., Kotsyfakis M., Kovárová Z., Sanda M., Majtán J., Grunclová L., Horká H., Andersen J.F. (2010). Crystal structure and functional characterization of an immunomodulatory salivary cystatin from the soft tick *Ornithodoros moubata*. Biochem. J..

[35] He B., Cai G., Ni Y., Li Y., Zong H., He L. (2011). Characterization and expression of a novel cystatin gene from Schistosoma japonicum. Mol. Cell. Probes..

[36] Wang Y., Yu X., Cao J., Zhou Y., Gong H., Zhang H., Li X., Zhou J. (2015). Characterization of a secreted cystatin from the tick *Rhipicephalus haemaphysaloides*. Exp. Appl. Acarol..

[37] Wang Y., Zhou Y., Gong H., Cao J., Zhang H., Li X., Zhou J. (2015). Functional characterization of a cystatin from the tick *Rhipicephalus haemaphysaloides*. Parasites Vectors.

[38] Wang Y., Wu L., Liu X., Wang S., Ehsan M., Yan R., Song X., Xu L., Li X. (2017). Characterization of a secreted cystatin of the parasitic nematode *Haemonchus contortus* and its immune-modulatory effect on goat monocytes. Parasites Vectors.

[39] Sun T., Wang F., Pan W., Wu Q., Wang J., Dai J. (2018). An Immunosuppressive Tick Salivary Gland Protein DsCystatin Interferes With Toll-Like Receptor Signaling by Downregulating TRAF6. Front. Immunol..

[40] Wei N., Lin Z., Xu Z., Gong H., Zhang H., Zhou Y., Cao J., Li G., Zhou J. (2019). Immunosuppressive effects of tick protein RHcyst-1 on murine bone marrow-derived dendritic cells. Parasites Vectors.

[41] Kotál J., Stergiou N., Buša M., Chlastáková A., Beránková Z., Řezáčová P., Langhansová H., Schwarz A., Calvo E., Kopecký J. (2019). The structure and function of Iristatin, a novel immunosuppressive tick salivary cystatin. Cell. Mol. Life Sci..

[42] Kotál J., Buša M., Urbanová V., Řezáčová P., Chmelař J., Langhansová H., Sojka D., Mareš M., Kotsyfakis M. (2021). Mialostatin, a Novel Midgut Cystatin from *Ixodes ricinus* Ticks: Crystal Structure and Regulation of Host Blood Digestion. Int. J. Mol. Sci..

[43] Ramirez Merlano J.A., Almeida D.V. (2022). Heterologous Production and Evaluation of the Biological Activity of Cystatin-B From the Red Piranha *Pygocentrus nattereri*. Front. Genet..

[44] Martins L.A., Buša M., Chlastáková A., Kotál J., Beránková Z., Stergiou N., Jmel M.A., Schmitt E., Chmelař J., Mareš M. (2023). Protease-bound structure of Ricistatin provides insights into the mechanism of action of tick salivary cystatins in the vertebrate host. Cell. Mol. Life Sci..

[45] Zhou J., Ueda M., Umemiya R., Battsetseg B., Boldbaatar D., Xuan X., Fujisaki K. (2006). A secreted cystatin from the tick *Haemaphysalis longicornis* and its distinct expression patterns in relation to innate immunity. Insect Biochem. Mol. Biol..

[46] Bennett M.J., Schlunegger M.P., Eisenberg D. (1995). 3D domain swapping: A mechanism for oligomer assembly. Protein Sci..

[47] Gronenborn A.M. (2009). Protein acrobatics in pairs—Dimerization via domain swapping. Curr. Opin. Struct. Biol..

[48] Liu L., Gronenborn A.M. (2012). 3.8 Protein and Nucleic Acid Folding: Domain Swapping in Proteins. Compr. Biophys..

[49] Zerovnik E., Turk V., Waltho J.P. (2002). Amyloid fibril formation by human stefin B: Influence of the initial pH-induced intermediate state. Biochem. Soc. Trans..

[50] Lin Y.M., Liu H.L., Zhao J.H., Huang C.H., Fang H.W., Ho Y., Chen W.Y. (2007). Molecular dynamics simulations to investigate the domain swapping mechanism of human cystatin C. Biotechnol. Prog..

[51] Shingate P., Warwicker J., Sowdhamini R. (2015). Energetic Calculations to Decipher pH-Dependent Oligomerization and Domain Swapping of Proteins. PLoS ONE.

[52] Wojciechowska D., Taube M., Rucińska K., Maksim J., Kozak M. (2022). Oligomerization of Human Cystatin C-An Amyloidogenic Protein: An Analysis of Small Oligomeric Subspecies. Int. J. Mol. Sci..

[53] Perlenfein T.J., Mehlhoff J.D., Murphy R.M. (2017). Insights into the mechanism of cystatin C oligomer and amyloid formation and its interaction with β-amyloid. J. Biol. Chem..

[54] Kolodziejczyk R., Michalska K., Hernandez-Santoyo A., Wahlbom M., Grubb A., Jaskolski M. (2010). Crystal structure of human cystatin C stabilized against amyloid formation. FEBS J..

[55] Staniforth R.A., Giannini S., Higgins L.D., Conroy M.J., Hounslow A.M., Jerala R., Craven C.J., Waltho J.P. (2001). Three-dimensional domain swapping in the folded and molten-globule states of cystatins, an amyloid-forming structural superfamily. EMBO J..

[56] Busa M., Mares M. (2024). Crystal Structure of Amacstatin-1, a Cystatin from the Hard Tick *Amblyomma maculatum*. https://www.wwpdb.org/pdb?id=pdb_00008r28.

[57] Busa M., Mares M. (2024). Crystal Structure of Domain Swapped Dimer of Amacstatin-2, a Cystatin from the Hard Tick *Amblyomma maculatum*. https://www.wwpdb.org/pdb?id=pdb_00008r29.

[58] Hong W., Cheng Z., Xu Z., Zhong S., Liu X., Dibo N., Dai Z., Lin Y., Lai W., Jia H. (2026). Crystal structure of *Echinococcus multilocularis* cystatin B reveals a novel feature in classical stefins. Sci. Rep..

[59] Bartošová-Sojková P., Dobai T., Kyslík J., Havlíčková P., Vancová M., Picard-Sánchez M.A., Qadiri S.S.N., Martins L.A., Urbanová V., Kosakyan A. (2026). Smolstatin1, a unique cystatin-like stefin of *Sphaerospora molnari*, is essential for parasite development. Curr. Res. Microb. Sci..

[60] Molari W.S., Jmel M.A., Assis J.B., Frazão-Silva A., Bernardi J.M., Huamanrayme G., Medina J.M., Esteves E., Antão S.C., Costa G.C.A. (2025). Amblyostatin-1, the first salivary cystatin with host immunomodulatory and anti-inflammatory properties from the Neotropical tick *Amblyomma sculptum*, vector of Brazilian spotted fever. Front. Immunol..

[61] Wei N., Lin Z., Xu Z., Cao J., Zhou Y., Zhang H., Gong H., Zhou J., Li G. (2018). A Tick Cysteine Protease Inhibitor RHcyst-1 Exhibits Antitumor Potential. Cell. Physiol. Biochem..

[62] Langhansová H., Beránková Z., Khanna R., Kotál J., Kotsyfakis M., Palus M., Lieskovská J. (2025). Tick salivary cystatin Iristatin limits the virus replication in skin of tick-borne encephalitis virus-infected mice. Parasitol. Res..

[63] Bennett M.J., Choe S., Eisenberg D. (1994). Domain swapping: Entangling alliances between proteins. Proc. Natl. Acad. Sci. USA.

[64] Schlunegger M.P., Bennett M.J., Eisenberg D. (1997). Oligomer formation by 3D domain swapping: A model for protein assembly and misassembly. Adv. Protein Chem..

[65] Yang S., Cho S.S., Levy Y., Cheung M.S., Levine H., Wolynes P.G., Onuchic J.N. (2004). Domain swapping is a consequence of minimal frustration. Proc. Natl. Acad. Sci. USA.

[66] Yang S., Levine H., Onuchic J.N. (2005). Protein oligomerization through domain swapping: Role of inter-molecular interactions and protein concentration. J. Mol. Biol..

[67] Zalar M., Golovanov A.P. (2019). New Disulphide Bond in Cystatin-Based Protein Scaffold Prevents Domain-Swap-Mediated Oli-gomerization and Stabilizes the Functionally Active Form. ACS Omega.

[68] Szymańska A., Jankowska E., Orlikowska M., Behrendt I., Czaplewska P., Rodziewicz-Motowidło S. (2012). Influence of point mutations on the stability, dimerization, and oligomerization of human cystatin C and its L68Q variant. Front. Mol. Neurosci..

[69] Rodziewicz-Motowidło S., Wahlbom M., Wang X., Lagiewka J., Janowski R., Jaskólski M., Grubb A., Grzonka Z. (2006). Checking the conformational stability of cystatin C and its L68Q variant by molecular dynamics studies: Why is the L68Q variant amyloi-dogenic?. J. Struct. Biol..

[70] Liu H.L., Lin Y.M., Zhao J.H., Hsieh M.C., Lin H.Y., Huang C.H., Fang H.W., Ho Y., Chen W.Y. (2007). Molecular dynamics simulations of human cystatin C and its L68Q varient to investigate the domain swapping mechanism. J. Biomol. Struct. Dyn..

[71] Esteves E., Maruyama S.R., Kawahara R., Fujita A., Martins L.A., Righi A.A., Costa F.B., Palmisano G., Labruna M.B., Sá-Nunes A. (2017). Analysis of the Salivary Gland Transcriptome of Unfed and Partially Fed *Amblyomma sculptum* Ticks and Descriptive Proteome of the Saliva. Front. Cell. Infect. Microbiol..

[72] Portaro F.C., Cezari M.H., Juliano M.A., Juliano L., Walmsley A.R., Prado E.S. (1997). Design of kallidin-releasing tissue kallikrein inhibitors based on the specificities of the enzyme’s binding subsites. Biochem. J..

