# Supplementary material for: Environment-Dependent Dimerization as a Functional Switch in Leech Cystatin CysHv
Source: Toxins (Basel). 2026 Jul 10;18(7):300. doi: 10.3390/toxins18070300 (PMC13418211; doi:10.3390/toxins18070300)
Supplement: Supplementary file 1 [file toxins-18-00300-s001.zip › toxins-4387289-supplementary.pdf]

Supplementary Materials

Environment-Dependent Dimerization as a Functional Switch in Leech Cystatin CysHv

Melissa Regina Fessel [1], Ana Marisa Chudzinski-Tavassi [1,2] and Fernanda Faria [1,*]

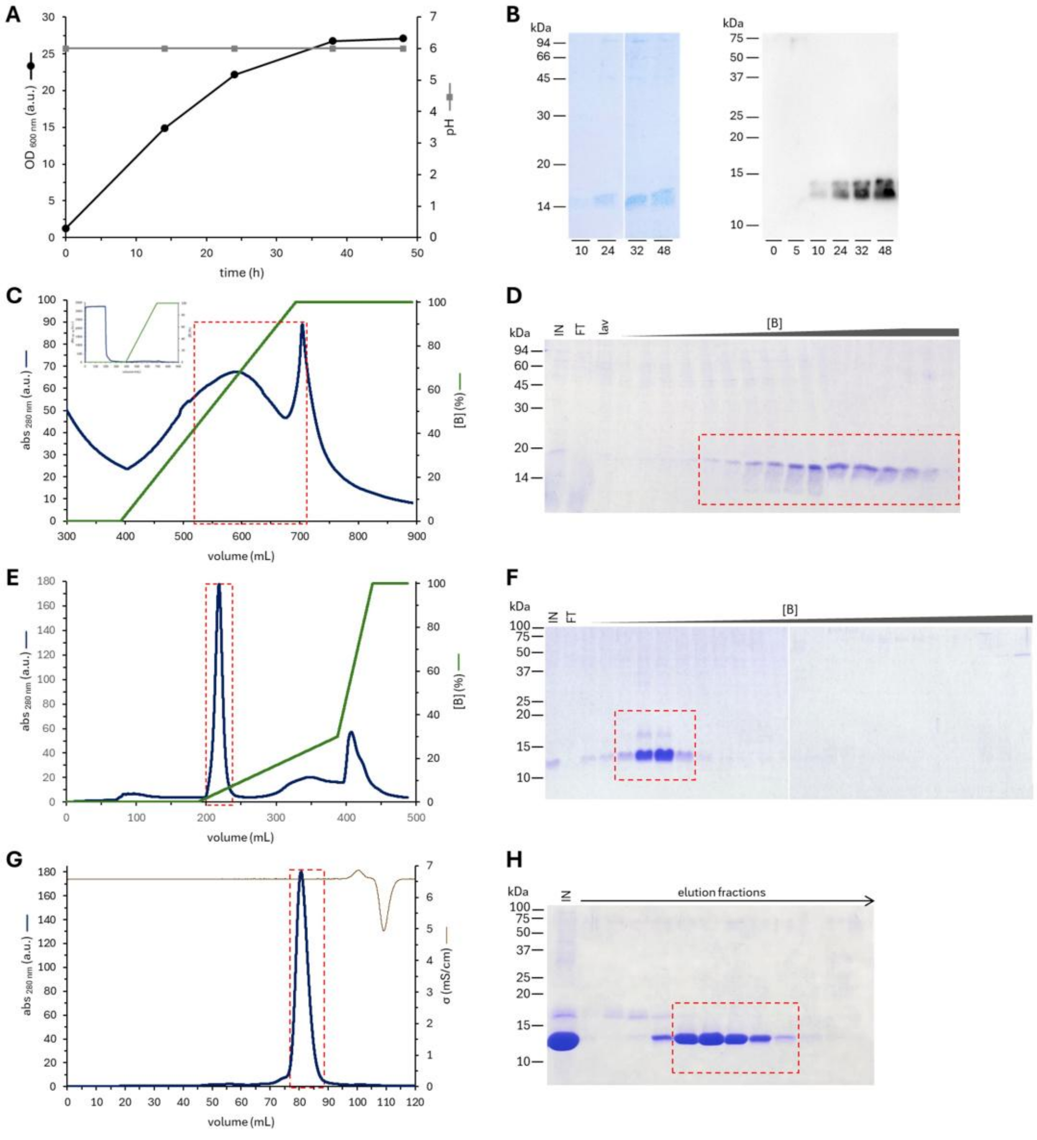

Figure S1. Representative results of CysHv expression and purification scheme. (A) Growth curve of a Pichia pastoris X-33 clone secreting CysHv in BMMY medium at 30 °C, 250 rpm, with daily addition of 0.5% methanol for 48 h, monitoring OD600 and culture pH. (B) At the indicated time points, culture supernatant aliquots were analyzed by 15% SDS-PAGE with Coomassie blue staining (left) and by western blot using an in-house anti-CysHv antibody (right). Molecular mass markers are shown in kDa. (C) Clarified supernatant was prepared by ammonium sulfate addition and subjected to hydrophobic interaction chromatography (HIC) on a HiTrap PHE HS FF column. (D) 15% SDS-PAGE Coomassie blue-stained gel of HIC fractions. (E) The protein pool was desalted and processed by ion-exchange chromatography (IEX) on a HiTrap Q FF column. (F) 15% SDS-PAGE Coomassie blue-stained gel of IEX fractions. (G) The IEX protein pool was concentrated and subjected to size-exclusion chromatography (SEC) on a Spx75 16/600 column. (H) 15% SDS-PAGE Coomassie blue-stained gel of SEC fractions. All chromatograms display absorbance at 280 nm (a.u.) as a function of elution volume and either buffer B concentration or conductivity. Dashed boxes indicate pooled fractions containing CysHv. Further methodological details are provided in the Methods section.

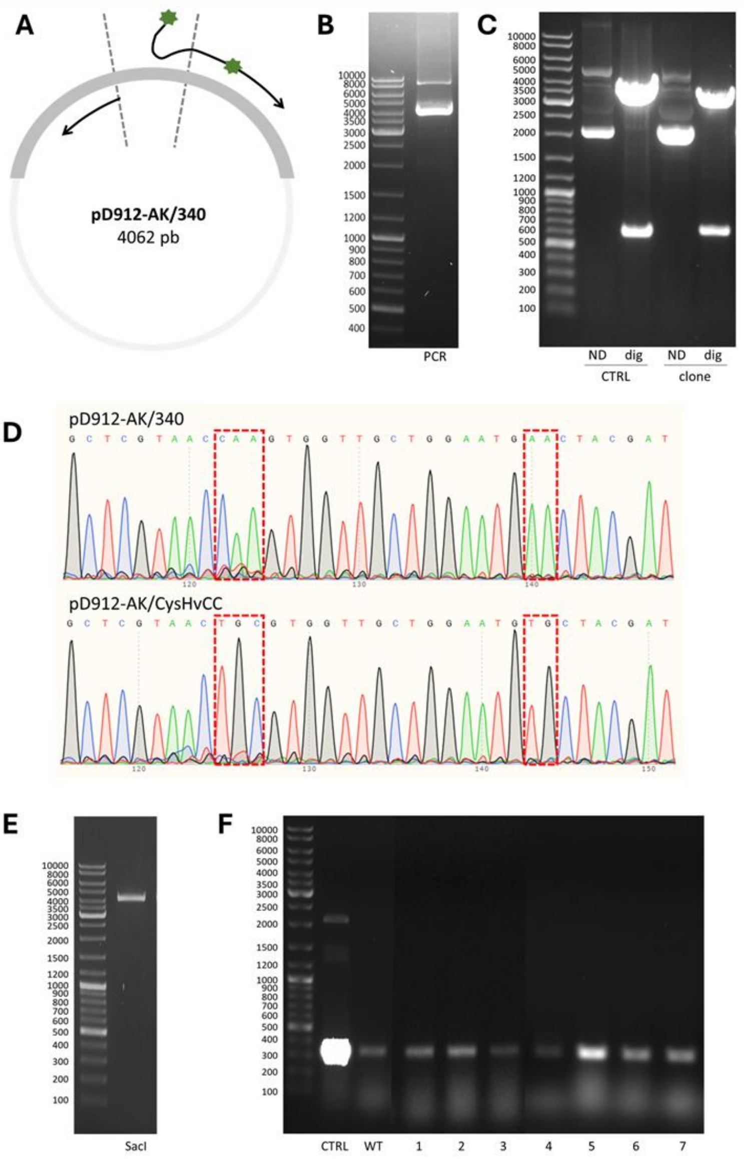

Figure S2. Obtention of double mutant CysHvCC. A single mutagenic primer was designed to mutate CysHv gene (3 and 2 nucleotides, 15 bases apart), using Phusion polymerase, and the parental plasmid (pD912-AK/340) as template, in a mutagenic PCR (A). PCR product (B) was DnpI digested to remove residual pD912-AK/340, transformed into *E. coli* DH5α, and positive clones were searched using restriction analysis. (CTRL = pD912-AK/340; clone = positive clone, pD912-AK/CysHvCC) (C). Confirmation of site-directed mutagenesis was performed by nucleotide sequencing analysis (D). On the top, chromatogram showing the region of interest from the parental plasmid (pD912-AK/340). On the bottom, the region of interest from the positive clone (pD912-AK/CysHvCC). The dashed-red line boxes indicate original (top) and mutated (bottom) nucleotides. For integration into *P. pastoris* X-33 genome, pD912-AK/CysHvCC was linearized with SacI (E), and transformed into fresh competent yeast cells by electroporation. Transformed zeocin-resistent *P. pastoris* cells were screened for positive integrants using colony PCR (F) (CTRL = pD912-AK/340; WT = pD912-AK/340-integrated yeast; 1-7, positive pD912-AK/CysHvCC-integrated clones).

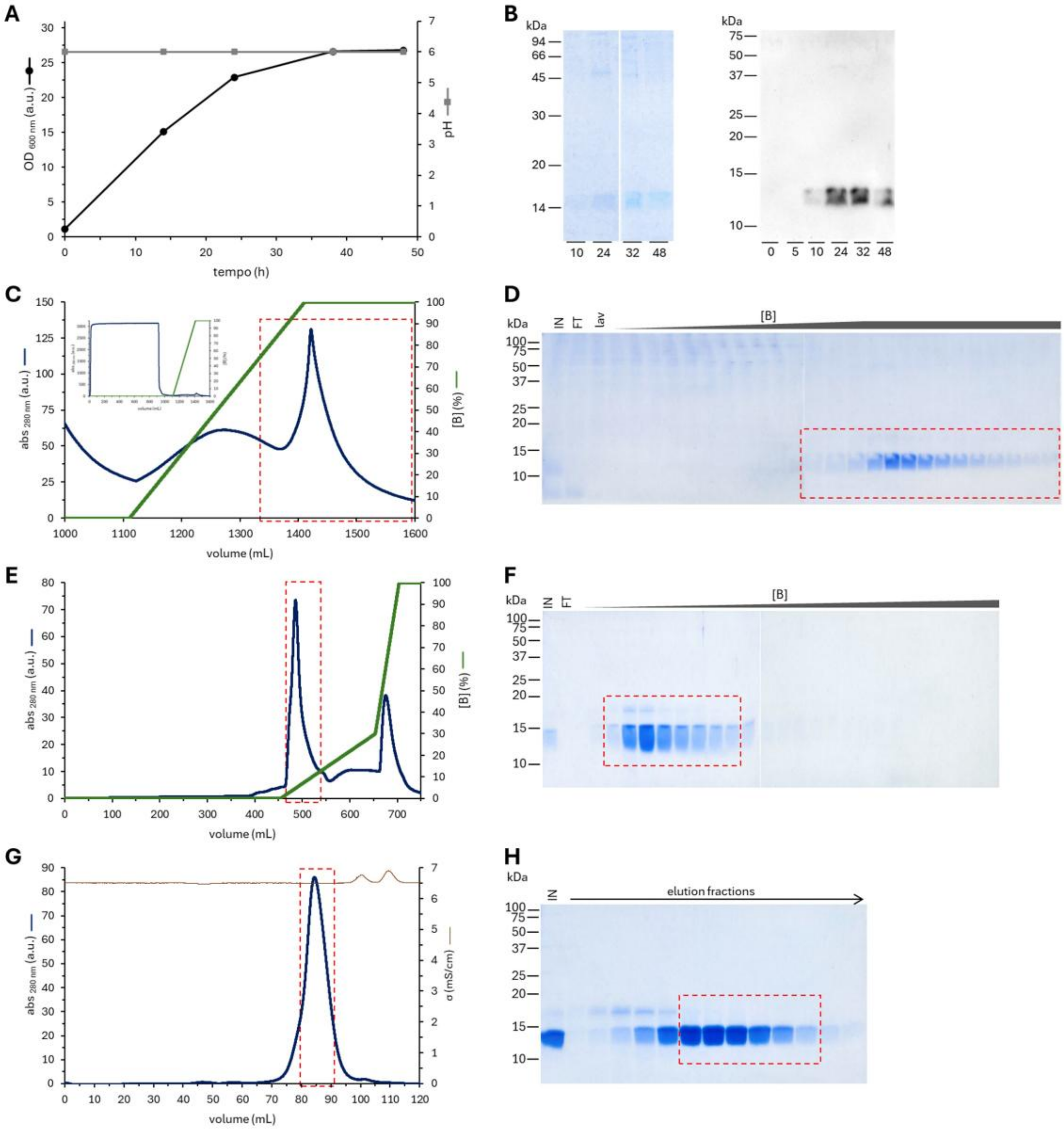

Figure S3. Representative expression and purification of the CysHv double mutant (CysHvCC). (A) Growth curve of a Pichia pastoris X-33 clone secreting CysHvCC in BMMY medium at 30 °C, 250 rpm, with daily addition of 0.5% methanol for 48 h, monitoring OD600 and culture pH. (B) At the indicated time points, culture supernatant aliquots were analyzed by 15% SDS-PAGE with Coomassie blue staining (left) and by western blot using an in-house anti-CysHv antibody (right). Molecular mass markers are shown in kDa. (C) Clarified supernatant was prepared by ammonium sulfate addition and subjected to hydrophobic interaction chromatography (HIC) on a HiTrap PHE HS FF column. (D) 15% SDS-PAGE Coomassie blue-stained gel of HIC fractions. (E) The protein pool was desalted and processed by ion-exchange chromatography (IEX) on a HiTrap Q FF column. (F) 15% SDS-PAGE Coomassie blue-stained gel of IEX fractions. (G) The IEX protein pool was concentrated and subjected to size-exclusion chromatography (SEC) on a Spx75 16/600 column. (H) 15% SDS-PAGE Coomassie blue-stained gel of SEC fractions. All chromatograms display absorbance at 280 nm (a.u.) as a function of elution volume and either buffer B concentration or conductivity. Dashed boxes indicate pooled fractions containing CysHvCC. Further methodological details are provided in the Methods section.

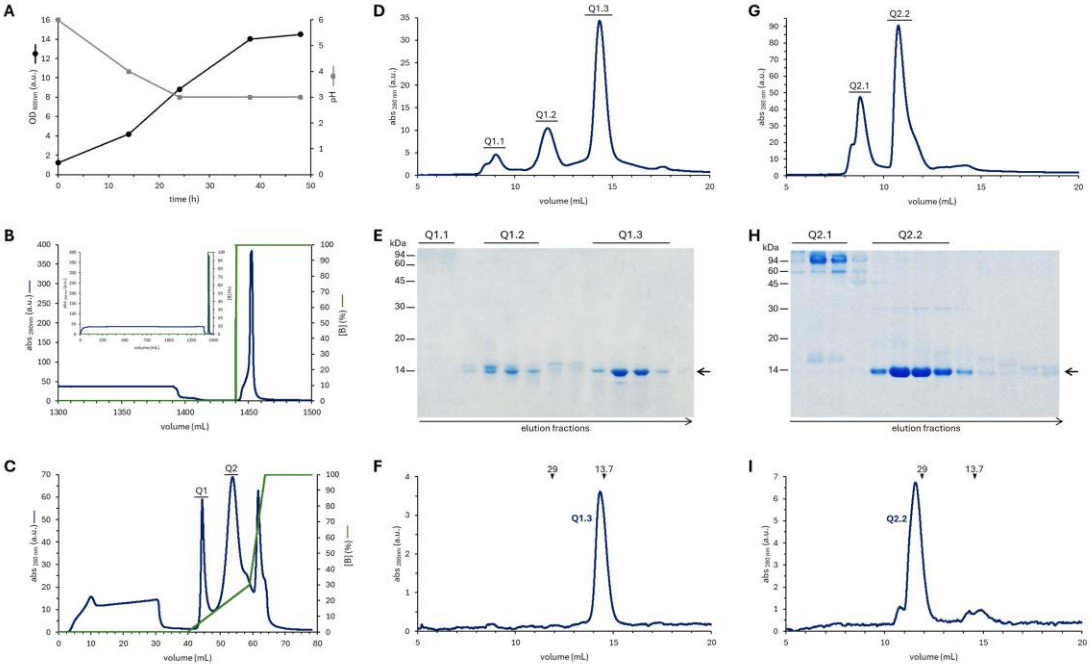

Figure S4. CysHv purified predominantly as a dimer under acidic conditions (pH 3). (A) Growth curve of a Pichia pastoris X-33 clone secreting CysHv in unbuffered MM medium at 30 °C and 250 rpm, with daily addition of 0.5% methanol for 48 h. Optical density (OD600) and culture pH were monitored. (B) Clarified culture supernatant (pH 3) was subjected to cation-exchange chromatography on a HiTrap SP FF column. (C) The protein pool was desalted and further separated by anion-exchange chromatography on a HiTrap Q FF column, yielding two main CysHv-containing peaks (Q1 and Q2). Individual IEX fractions were concentrated and subjected to size-exclusion chromatography (SEC) on a Superdex 75 10/300 column. (D–E) SEC fractionation of peak Q1 revealed three peaks (D), and analysis by 15% SDS-PAGE followed by Coomassie Blue staining indicated that CysHv was predominantly present in fraction Q1.3 (E). (F) SEC analysis of fraction Q1.3 is consistent with monomeric CysHv. (G–H) SEC fractionation of peak Q2 revealed two main peaks (G), and SDS-PAGE analysis indicated that CysHv was predominantly present in fraction Q2.2 (H). (I) SEC analysis of fraction Q2.2 is consistent with dimeric CysHv.

A

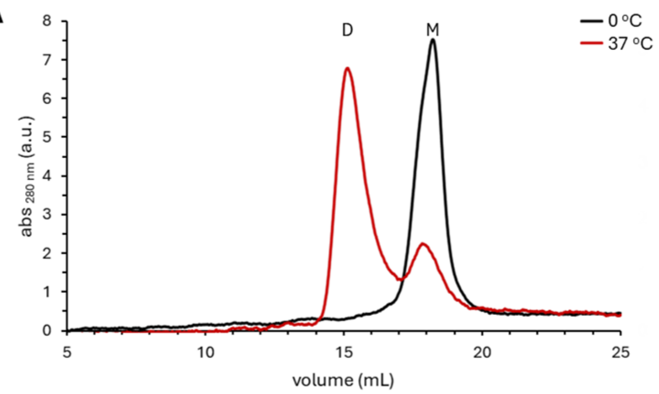

pH 3		
specie	oligomeric state (%)	
	0 °C	37 °C
monomer (M)	100	86.7
dimer (D)	0	13.3

B

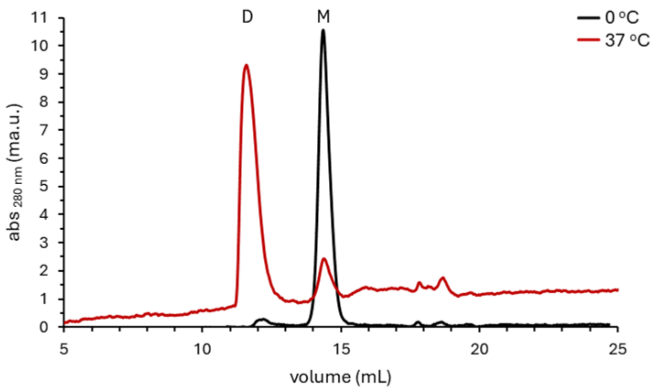

pH 8		
specie	oligomeric state (%)	
	0 °C	37 °C
monomer (M)	98.6	90.3
dimer (D)	1.4	9.7

Figure S5. Oligomeric state distribution of CysHv determined by SEC at pH 3 and pH 8. Freshly purified CysHv (0.4 g/L, pH 8) was buffer-exchanged into pH 3 and incubated for 48 h at the indicated temperatures. (A) Chromatographic profiles of samples analyzed by size-exclusion chromatography (SEC) using a Superdex 75 10/300 column equilibrated at pH 3 show altered elution profiles, suggesting non-specific interactions with the column under these conditions. Peak area integration was used to estimate the relative monomer and dimer content (%), as indicated on the right. (B) The same samples were analyzed by SEC at pH 8, revealing comparable distributions of oligomeric states.

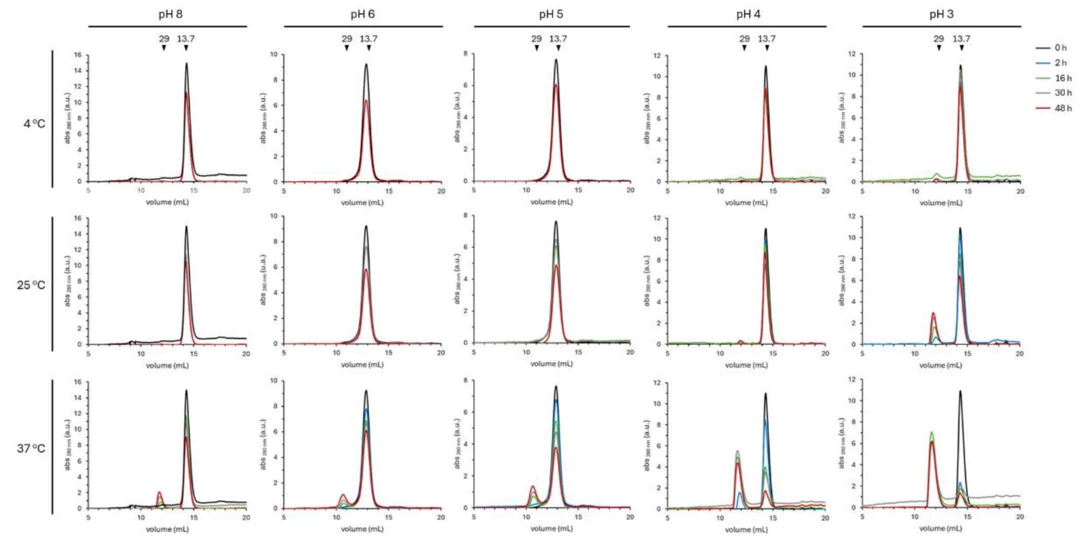

Figure S6. Representative SEC chromatograms showing time-, temperature-, and pH-dependent changes in the oligomeric distribution of CysHv. Freshly purified CysHv (pH 8) was buffer-exchanged into sodium citrate buffers (pH 6–3) and incubated at 0.4 g/L at 4 °C, 25 °C, or 37 °C, as indicated. Aliquots collected over time (0 to 48 h; legend, top right) were analyzed by size-exclusion chromatography (SEC) on a Superdex 75 10/300 column equilibrated at pH 8. For each pH condition, elution volumes of standard proteins are indicated (kDa). Monomer and dimer peak areas were integrated, and their sum was normalized to 100% for oligomeric state quantification. For clarity, only chromatograms displaying observable changes over time are shown.

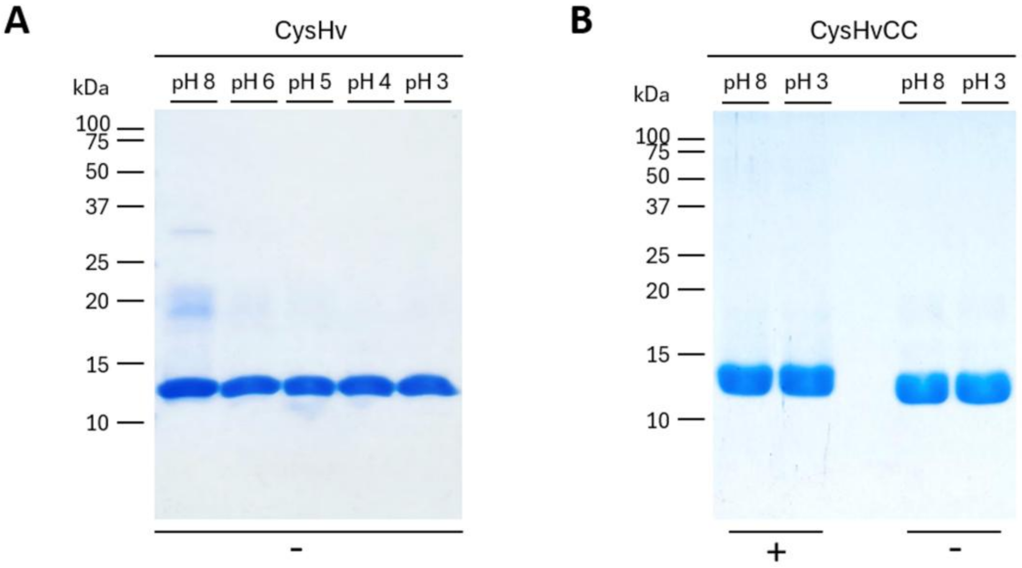

Figure S7. Non-reducing electrophoretic profiles of CysHv and CysHvCC submitted to thermal stress under different pH conditions. (A) SDS-PAGE (15%) analysis of CysHv incubated for 72 h at the indicated pH values under non-reducing conditions (-). (B) SDS-PAGE (15%) analysis of CysH-vCC incubated for 48 h at 37 °C at the indicated pH values under reducing (+) or non-reducing conditions (-). Gels were stained with Coomassie blue. Molecular mass standards are indicated on the left, in kDa.

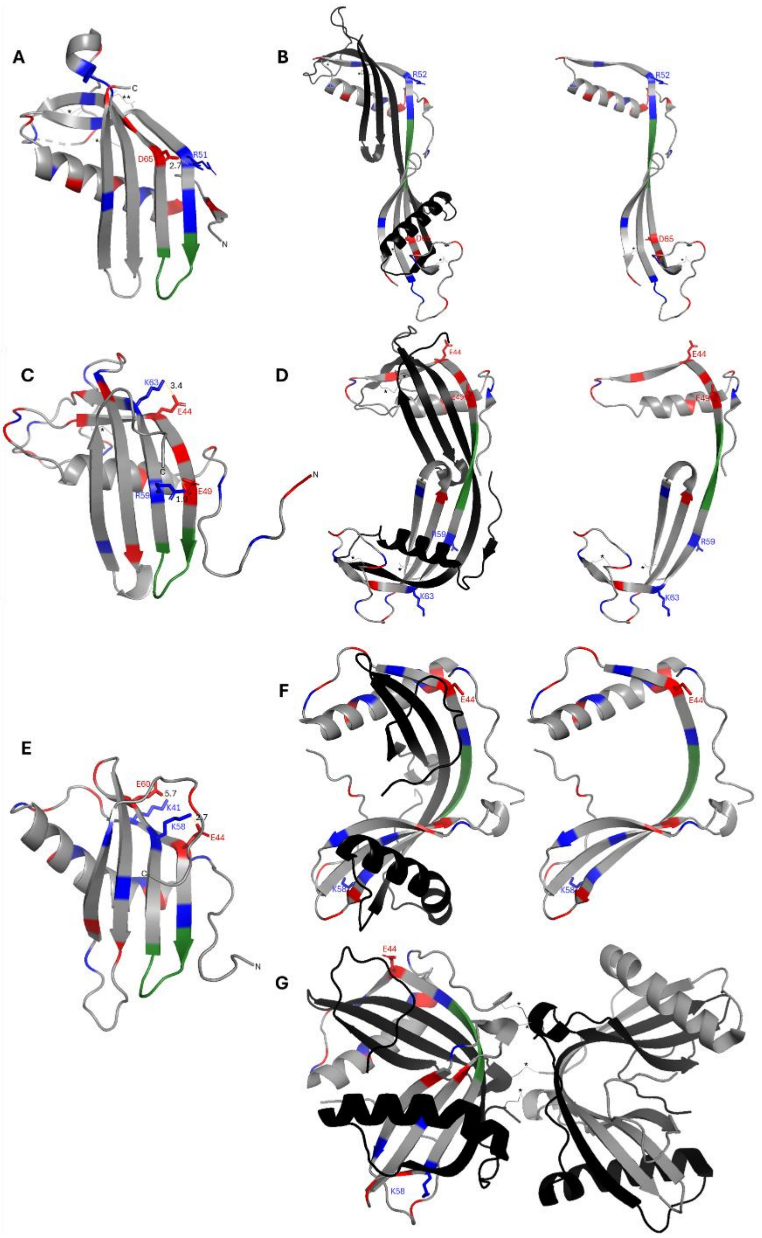

Figure S8. Additional examples of the spatial arrangement of charge-complementary residues in L1 loop-flanking β-strands across cystatins crystallized as domain-swapped dimers. (A-B) Human cystatin C monomer (PDB: 3GAX) stabilized by an engineered disulfide bond (indicated by **), showing a pair of oppositely charged residues in close spatial proximity within the β-strands flanking the L1 loop (A), which become spatially separated upon domain-swapped dimerization (PDB: 1TIJ) (B). The domain-swapped structure is shown with both chains colored black and gray (left) and, for clarity, as an individual chain (right). (C-D) Amacstatin 2 modeled as a monomer (C), revealing two pairs of oppositely charged residues in close spatial proximity within the β-strands flanking the L1 loop, which become separated in the domain-swapped dimer (PDB: 8R29) (D). The domain-swapped structure is shown as described above. (E-G) EmCystatin-B modeled as a monomer (E), showing one pair of oppositely charged residues in close spatial proximity within the β-strands flanking the L1 loop, which become separated in the domain-swapped dimer (derived from PDB: 9UOZ) (F). The domain-swapped dimer is shown as described above. (G) Crystal structure of EmCystatinB forming a tetramer composed of two domain-swapped dimers linked by disulfide bonds (PDB: 9UOZ).

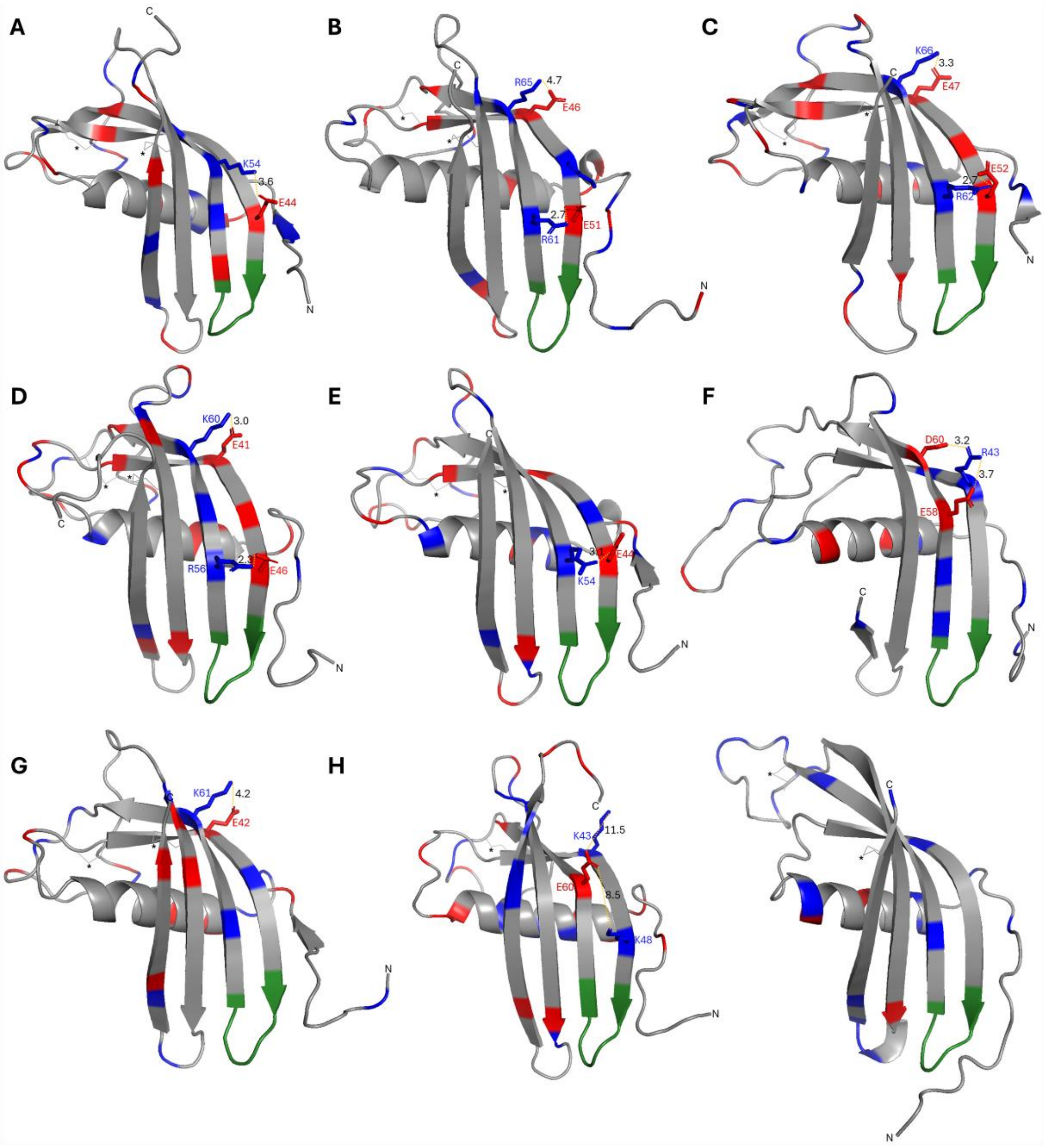

Figure S9. Additional examples of the spatial arrangement of charge-complementary residues in L1 loop-flanking β-strands across homologous secreted cystatins from hematophagous organisms. (A) Iristatin (PDB: 5o46) (B), Ricistatin (AlphaFold 2 model), (C) Amacstatin 1 (PDB: 8r28), (D) RHcyst2 (Robetta model), (E) DsCystatin (Robetta model), (F) HlSC-1 (Robetta model), (G) Om-cystatin 1 (AlphaFold2 model), (H) HcCyst-3 (Robetta model), and (I) Amblyostatin 1 (AlphaFold2 model). Monomeric structures were obtained from the Protein Data Bank or generated as indicated. All structures are shown in cartoon representation, with disulfide bonds depicted as lines (indicated by *) and the inhibitory L1 loop highlighted in green. Positively and negatively charged residues are shown in blue and red, respectively. Charge-complementary residues located in the β-strands flanking the L1 loop are represented as sticks, and distances between oppositely charged side-chain atoms are indicated in angstroms. N- and C-termini are indicated. In most analyzed structures, oppositely charged residues are positioned in close spatial proximity and are consistent with potential hydrogen bond interactions as predicted by PropKa (see Table S1). In contrast, in Om-cystatin 1 and in HcCyst-3 these residues are more distant, and Amblyostatin 1 no such pairs were identified in equivalent positions.

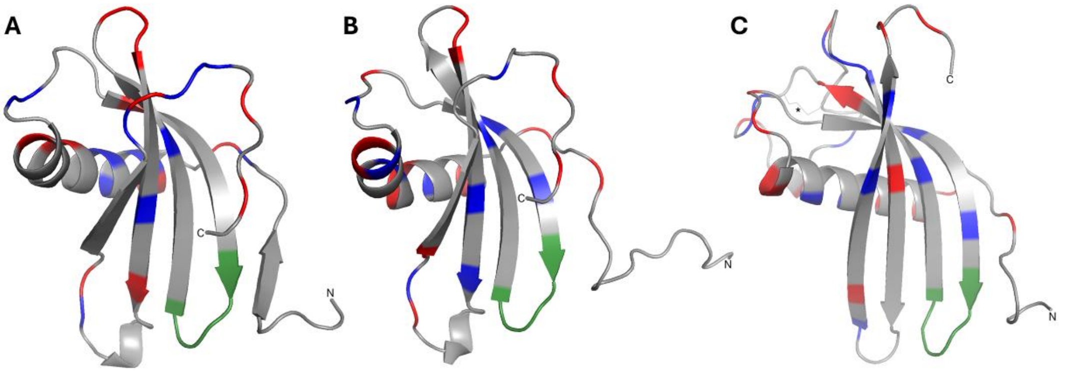

Figure S10. Additional intracellular cystatins showing absence of proximal oppositely charged residues in L1 loop-flanking β-strands. (A) SjCystatin, (B) PnCystB, and (C) HcCys1. Structural models revealed no oppositely charged residues in close spatial proximity within the β-strands flanking the L1 loop. All structures are shown in cartoon representation, with disulfide bonds indicated as lines (*) and the inhibitory L1 loop highlighted in green. Positively and negatively charged residues are shown in blue and red, respectively.

Table S1. PropKa-based analysis of charge-complementary residues in L1 loop-flanking β-strands of selected cystatins. Proteins were classified as intracellular or extracellular based on the absence or presence of a signal peptide. Structural models were obtained from experimentally determined structures (PDB) or predicted using AlphaFold2 or Robetta, as indicated, and analyzed using PROPKA. Residues located in the β-strands flanking the L1 loop were inspected for oppositely charged side chains. When present, PROPKA-predicted sidechain interaction energies (in parentheses) are reported and were consistently classified as "sidechain hydrogen bond". These values represent estimated electrostatic contributions and do not necessarily indicate stable hydrogen bonds in solution. In some cases, oppositely charged residues are present but not predicted to interact, consistent with spatial separation beyond interaction range. Absence of values indicates that no interaction was predicted under the analyzed conditions. Predicted pKa values for individual residues are also provided. For CysHv, additional charge-complementary residues pairs are also presented (indicated by *).

Protein name	Species	Subcellular localization	Structure source		PropKa	
			dimer	monomer	Sidechain Hydrogen Bond	Predicted pKa
CysHv	*Haementeria Vizottoi*	extracellular	Swiss model	Robetta	D57-R47 (-0.79)<br>*D19-K23 (-1.20)<br>*E108-R110 (-0.79)	D57: 2.63<br>R47: 12.50
hCC	*Homo sapiens*	extracellular	PDB: 1tij	PDB: 3gax	D65-R51 (-0.80)	D65: 2.69<br>R51: 11.94
Sialostatin L	*Ixodes scapularis*	extracellular	PDB: 4zm8	Alphafold2	E45-K64 (-0.21)<br>E50-R60 (-2.40)	E45: 4.50<br>K64: 10.43<br>E50: 2.42<br>R60: 11.66
Amacstatin 2	*Amblyomma Maculatum*	extracellular	PDB: 8r29	Alphafold2	E49-R59 (-2.40)	E44: 4.57<br>K63: 10.50<br>E49: 2.45<br>R59: 11.45
EmCystatin	*Echinococcus Multilocularis*	intracellular	PDB: 9uoz	Alphafold2	E44-K58 (-0.80)	E44: 3.98<br>K58: 9.80<br>E60: 4.63<br>K41: 10.50
Amacstatin 1	*Amblyomma Maculatum*	extracellular	-	PDB: 8r28	E47-K66 (-0.60)<br>E52-R62 (-0.80)	E47: 4.04<br>K66: 10.22<br>E52: 4.50<br>R62: 11.66
Amblyostatin 1	*Amblyomma Sculptum*	extracellular	-	Alphafold2	-	-
DsCystatin	*Dermacentor Silvarum*	extracellular	-	Robetta	E44-R54 (-0.72)	E44: 3.27<br>R54: 11.80
Om-cystatin 2	*Ornithodoros Moubata*	extracellular	-	PDB: 3l0r	E59-K42 (-0.50)	E59: 3.79 K42: 10.08
Ricistatin	*Ixodes ricinus*	extracellular	-	Alphafold2	E51-R61 (-0.80)	E46: 4.57<br>K65: 10.36<br>E51: 3.32<br>R61: 11.66
RHcyst-2	*Rhiphicephalus Haemaphysaloides*	extracellular	-	Alphafold2	E41-K60 (-0.80) E46-R56 (-0.80)	E41: 3.91<br>K60: 10.50<br>E46: 4.05<br>R56: 12.50
HlSC-1	*Haemaphysalis Longicornis*	extracellular	-	Robetta	E58-R43 (-0.80) D60-R43 (-0.66)	E58: 3.77<br>R43: 12.01<br>D60: 2.35
Sialostatin L2	*Ixodes scapularis*	extracellular	-	PDB: 3lh4	E50-R60 (-2.40)	E50: 2.52<br>R60: 11.73
Iristatin	*Ixodes ricinus*	extracellular	-	PDB: 5o46	E44-K54 (-0.19)	E44: 4.41<br>K54: 10.08

Mialostatin	*Ixodes ricinus*	extracellular	-	PDB: 6ztk	-	E50: 4.64<br>K62: 10.43<br>K64: 10.43
HCcyst-3	*Haemonchus contortus*	extracellular	-	Robetta	-	E60: 3.96<br>K43: 10.08<br>K48: 10.50
Om-cystatin 1	*Ornithodoros moubata*	extracellular	-	Robetta	-	E42: 4.57<br>K61: 10.29
SjCystatin	*Schistosoma japonicum*	intracellular	-	Robetta	-	-
PnCystB	*Pygocentrus natterei*	intracellular	-	Robetta	-	-
TtcysB	*Theromyzon tessulatum*	intracellular	-	Robetta	-	-
HcCys1	*Haemonchus contortus*	intracellular	-	Robetta	-	-
RHcyst-1	*Rhiphicephalus haemaphysaloides*	intracellular	-	Robetta	-	-
Bmcystatin	*Boophilus microplus*	intracellular	-	Robetta	-	-
